# Resveratrol for Post-COVID-19 Cognitive Dysfunction: From Molecular Basis to Clinical Translation

**DOI:** 10.3390/molecules31203747

**Published:** 2026-10-19

**Authors:** Margarita L. Martinez-Fierro, Samantha Gonzalez-Delgado, Israel A. Solis-Hernandez, Maria del R. Rocha-Pizaña, Ana J. Mendez-Gonzalez, Idalia Garza-Veloz

**Affiliations:** 1 Molecular Medicine Laboratory, Academic Unit of Human Medicine and Health Sciences, Universidad Autónoma de Zacatecas, Zacatecas-Guadalajara km. 6, Ejido La Escondida, Zacatecas 98160, Mexico; samanthagonzalez407@gmail.com (S.G.-D.); 36176850@uaz.edu.mx (I.A.S.-H.); 20205406@uaz.edu.mx (A.J.M.-G.); 2 Escuela de Ingeniería y Ciencias, Tecnológico de Monterrey, Av. Eugenio Garza Sada 2501, Monterrey 64849, Mexico; mrochap@tec.mx

**Keywords:** SARS-CoV-2, post-COVID-19 condition, long COVID, cognitive dysfunction, blood–brain barrier, neuroinflammation, resveratrol, SIRT1, oxidative stress, synaptic plasticity

## Abstract

Post-COVID-19 condition can include persistent cognitive symptoms, commonly described as impaired attention, memory difficulties, slowed processing, and “brain fog.” Clinical and translational evidence implicates systemic inflammation, neurovascular dysfunction, blood–brain barrier disruption, glial inflammatory responses, oxidative stress, and altered synaptic or network function; their relative contributions remain uncertain and may differ across patient subgroups. Resveratrol is a polyphenolic compound that modifies inflammatory, redox, metabolic, endothelial, and antiviral pathways in experimental systems. This focused narrative review examines whether those actions provide a mechanistic basis for the use of resveratrol in post-COVID-19 cognitive dysfunction. Evidence was considered according to biological proximity to the intended indication, distinguishing human post-acute sequelae of SARS-CoV-2 infection (PASC) studies from acute-COVID-19, non-COVID-19 clinical, animal, organoid, and cell-based evidence. The review identifies candidate neurovascular, immune, and metabolic processes, evaluates where resveratrol evidence intersects with those processes, and distinguishes molecular observations from clinical questions that remain to be tested for to define the therapeutic potential of using resveratrol to treat post-COVID-19 cognitive impairment.

## 1. Introduction

SARS-CoV-2 infection can be followed by persistent symptoms involving multiple organ systems. Cognitive complaints are among the most functionally consequential manifestations of post-COVID-19 condition. The term “brain fog” encompasses a spectrum of experiences, including reduced mental clarity, slowed processing, impaired attention, forgetfulness, working-memory difficulties, fatigue, sleep disturbances, and affective symptoms.

The World Health Organization defines post-COVID-19 condition as the presence of symptoms that usually occur 3 months after infection, persist for at least 2 months, and cannot be explained by an alternative diagnosis [1]. This definition identifies a clinical syndrome characterized by biological heterogeneity. Population studies and systematic reviews have documented measurable differences in attention, executive function, processing speed, memory, and related cognitive domains following SARS-CoV-2 infection, with variation in magnitude and duration across cohorts [2,3]. Cognitive performance after infection reflects the contribution of multiple clinical and individual factors, including acute illness severity, hypoxemia, hospitalization, intensive-care exposure, delirium, reinfection, vaccination history, premorbid cognitive reserve, depression, anxiety, sleep disorders, dysautonomia, fatigue, and medication effects.

Resveratrol (3,5,4′-trihydroxystilbene), a stilbene belonging to the polyphenol family, is a naturally occurring polyphenol originally isolated from the dried rhizome of *Veratrum grandiflorum* [4,5]. This compound has subsequently been identified in grape skins and wines, selected Vaccinium berries, preparations of Itadori tea, and cocoa-containing products [6,7,8,9].

Resveratrol exists in two stereoisomeric forms, *cis* and *trans*, with the trans isomer considered the most biologically active and responsible for most of its reported physiological effects [4,10]. Resveratrol has attracted interest because experimental studies report effects on inflammatory signaling, oxidative stress (OS), endothelial activation, mitochondrial homeostasis, and specific antiviral pathways [4,10,11]. These activities overlap with candidate processes implicated in some post-COVID-19 cognitive phenotypes. Their clinical relevance remains uncertain because oral parent-resveratrol exposure is limited by rapid metabolism. Pharmacologically relevant central nervous system (CNS) exposure has not been established in post-acute sequelae of SARS-CoV-2 infection (PASC), and no randomized trial has tested its cognitive benefit in the intended population. This focused narrative review examines the evidence linking post-COVID-19 cognitive dysfunction to neurovascular, neuroimmune, metabolic, and circuit-level alterations and then evaluates the directness and translational relevance of resveratrol evidence at each step. Studies reporting evidence of human PASC cognitive dysfunction were distinguished from acute-COVID-19, non-COVID-19 clinical, animal, organoid, and cell-based studies.

## 2. Material and Methods

### 2.1. Literature Identification and Selection

This focused narrative review was developed using an evidence-informed narrative approach, with the scale for the assessment of narrative review articles (SANRA) used as a guide for transparency, scientific reasoning, and presentation of data [12].

Studies were identified through searches of PubMed, Scopus, and Web of Science conducted through to May 2026. Search concepts combined terms related to “SARS-CoV-2” or “COVID-19”, “post-COVID-19 condition” or “long COVID”, “cognitive dysfunction” or “brain fog”, “neuroinflammation”, “neurovascular injury”, “blood–brain barrier dysfunction”, “synaptic plasticity”, “neurogenesis”, “angiogenesis”, and “resveratrol”. Reference lists of key reviews and primary studies were also examined to identify relevant reports not captured by the initial searches.

### 2.2. Inclusion Criteria

Priority was given to peer-reviewed human studies of post-COVID-19 cognitive impairment, neurovascular dysfunction, and blood–brain barrier (BBB) alteration; primary studies using SARS-CoV-2-relevant endothelial, neural, glial, organoid, or microphysiological models; randomized or controlled human studies of resveratrol; and primary mechanistic studies that directly examined pathways discussed in the review.

Studies of acute COVID-19, non-COVID-19 neurological disorders, animal models, organoids, and cell systems were included when they clarified a candidate mechanism or informed target selection, pharmacokinetics, or study design. However, these sources were not interpreted as direct evidence of clinical efficacy for post-COVID-19 cognitive dysfunction.

### 2.3. Evaluation and Prioritization of Evidence

Evidence was appraised according to study design, biological proximity to the intended clinical indication, exposure relevance, outcome relevance, and the extent to which a source could support causal inference. Direct human PASC evidence was distinguished from indirect human evidence, animal and organoid evidence, and in vitro or computational evidence.

Primary studies were used for quantitative, molecular, pharmacokinetic, safety, and intervention claims whenever available. Table 1 summarizes the evidence hierarchy applied throughout the manuscript. Because this review was not designed as a systematic review, no formal meta-analysis, duplicate independent screening, or standardized risk-of-bias assessment was performed. The approach may therefore be subject to selection bias and may not capture all relevant studies. These limitations were addressed by explicit evidence-level labeling and by avoiding efficacy or causal conclusions that exceed the directness of the available evidence.

## 3. Clinical Phenotype and Assessment of Post-COVID Cognitive Dysfunction

The clinical phenotype of post-COVID-19 cognitive dysfunction refers to an observable pattern of new, persistent, or worsening cognitive symptoms after SARS-CoV-2 infection, with or without objectively demonstrable deficits on standardized neuropsychological testing. It is a descriptive construct and identifies a clinically recognizable presentation considering that not all affected individuals share the same underlying mechanism.

Cognitive findings are variable across cohorts [2,3,13]. In a large cross-sectional, community-based online study, Hampshire et al. (2021) found lower performance across multiple cognitive domains among participants who had recovered from COVID-19 [13]. After adjustment for demographic characteristics, pre-existing medical disorders, tiredness, depression, and anxiety; the largest differences occurred among previously hospitalized participants. The study design was cross-sectional, exposure and symptoms were self-reported; therefore, the findings should be considered in that context. In smaller clinical cohorts selected for post-COVID-19 cognitive complaints, deficits have involved attention, processing speed, executive function, working and episodic memory, visuospatial processing, and naming, although their relative prominence differs among patients [14,15]. Figure 1 summarizes the neurological and cognitive symptom domains reported after COVID-19.

The term brain fog is useful as a patient-centered description of reduced mental clarity, slowed thinking, forgetfulness, and difficulty sustaining attention, but it is not a diagnostic entity and should not be treated as synonymous with objectively measured cognitive impairment. In the post-COVID-19 cohort studied by Delgado-Alonso et al., objective performance correlated with olfactory dysfunction, sleep quality, and, to a lesser extent, anxiety [15]. Conversely, in a small cohort of survivors of severe COVID-19 assessed after intensive-care admission, subjective cognitive complaints were associated with anxiety but not with neuropsychological performance [16]. Thus, cognitive complaints, an abnormal screening score, and impairment documented through a domain-specific neuropsychological battery are related but non-interchangeable outcomes.

Post-infection cognitive performance may also be shaped by acute disease and premorbid health. In a longitudinal cohort of 106 previously hospitalized patients, worse MoCA performance at six months was associated with greater acute respiratory severity and higher premorbid vulnerability [17]. Among survivors of intensive-care treatment, delirium, mechanical ventilation, sedation exposure, and inflammatory burden were associated with later neuropsychological outcomes, while cognitive reserve modified some of these relationships [16]. In older adults hospitalized with COVID-19, in-hospital delirium was also associated with cognitive impairment during the six months after discharge [18]. Fatigue, dysautonomia, depressive symptoms, and subjective memory complaints co-occurred in a neurological long-COVID-19 clinic cohort, but that self-report study could not establish that any of these symptoms causes objectively measured cognitive impairment [19].

According to the above, a clinically informative assessment should combine a structured history, domain-specific cognitive testing, and measures of functional impact with an evaluation of relevant concurrent symptoms and acute-illness exposures. Post-COVID-19 cognitive dysfunction is therefore best approached as a heterogenous clinical phenotype. This heterogeneity has direct implications for preclinical studies and therapeutic trials, which should define the cognitive phenotype objectively and account for competing contributors before intervention.

## 4. Mechanisms Relevant to Post-COVID-19 Cognitive Dysfunction

Post-COVID cognitive dysfunction emerges from interacting neurovascular, immune, metabolic, and circuit-level processes whose relative importance differs across clinical phenotypes. To preserve the evidence hierarchy, Section 4.1 summarizes neurovascular-unit and BBB biology and direct human PASC neurovascular evidence. Section 4.2, Section 4.3, Section 4.4 and Section 4.5 examine candidate mechanisms derived predominantly from acute COVID-19, post-mortem tissue, animal, organoid, and cell-based studies while identifying the limited PASC observations currently available in each area. Section 4.6 reports neuroanatomical and functional-network alterations in post-COVID-19 cognitive dysfunction [20,21,22].

### 4.1. The Blood–Brain Barrier: Structure, Function, and Vulnerability

The BBB is a regulated interface between the peripheral circulation and the CNS. Its anatomical and functional integrity depends on the neurovascular unit (NVU), which includes cerebral microvascular endothelial cells, pericytes, astrocytes, neurons, and extracellular-matrix components [23]. Brain endothelial cells combine tight junctions, low basal vesicular transport, and polarized transport systems to regulate molecular exchange between blood and the CNS [23]. Tight junctions are dynamic multimolecular assemblies of transmembrane and cytoplasmic proteins that also function as intracellular signaling platforms. Claudin-5, occludin, and junctional adhesion molecules form the junctional complex, and zonula occludens proteins connect these structures to the actin cytoskeleton. VE-cadherin (vascular endothelial cadherin)-based adherens junctions provide a complementary system of endothelial cell–cell adhesion and signaling. Their organization is dynamically responsive to inflammatory mediators, and disruption or phosphorylation of VE-cadherin can promote intercellular gap formation and increased endothelial permeability [24,25].

The endothelial monolayer rests on a basement membrane that contains collagen IV, laminins, nidogens, and heparan sulfate proteoglycans. Pericytes embedded in this matrix contribute to capillary stability, endothelial differentiation, perfusion, and barrier maintenance. Astrocyte end-feet provide metabolic, ionic, and trophic support, while neurons, microglia, oligodendroglia, and extracellular-matrix components shape local signaling. This organization favors a connection between the endothelial state and pericyte function, astrocytic support, cerebral perfusion, immune surveillance, and synaptic homeostasis [23]. The endothelial glycocalyx forms the blood-facing surface layer of the endothelium and contributes to endothelial barrier properties, inflammatory cell–endothelial interactions, and flow-dependent mechanotransduction. Its surface heparan sulfate proteoglycans also contribute to the anticoagulant properties of the vascular interface [26,27,28].

Molecular exchange at the BBB is governed by several coordinated mechanisms: (a) endothelial tight junctions restrict paracellular flux; (b) carrier-mediated systems supply essential nutrients, including glucose and large neutral amino acids; (c) receptor-mediated transcytosis permits selective transfer of ligands such as transferrin; and (d) ATP-dependent efflux transporters, including P-glycoprotein (P-gp), restrict brain exposure to many xenobiotics [29,30,31,32]. Immune surveillance at the BBB depends on regulated leukocyte–endothelial interactions. Adhesion molecules and chemokine-dependent signaling coordinate sequential leukocyte arrest, polarization, intraluminal crawling, and diapedesis across the brain endothelium; the relative contribution of individual molecules depends on endothelial activation and hemodynamic conditions [33,34]. The transport and trafficking functions have direct relevance because plasma exposure, neurovascular exposure, active-metabolite exposure, and CNS target engagement represent distinct measurements. In the same sense, carrier-mediated transport systems, efflux transporters, and receptor-mediated transcytosis shape the passage of endogenous substrates and therapeutic agents across the BBB. Their substrate specificity, directionality, and species-dependent activity are therefore central to translating experimental CNS-exposure data into human drug-development studies.

Accounting for species-specific differences in BBB transporter activity can further strengthen the translation of findings from animal models to human CNS exposure.

#### Neurovascular Dysfunction and Blood–Brain Barrier Disruption

In post-COVID-19 cognitive dysfunction, neurovascular biology provides an important clinical route through which systemic immune, endothelial, and coagulation-related signals may shape brain homeostasis.

The term “BBB disruption” encompasses several measurable processes, including changes in junctional organization, transcytosis, basement-membrane remodeling, endothelial injury, carrier or efflux activity, glycocalyx integrity, and leukocyte adhesion or migration. Matrix metalloproteinases (MMPs), particularly MMP-9, contribute to neurovascular-unit remodeling and BBB permeability by modifying extracellular-matrix and tight-junction organization [35].

In SARS-CoV-2 research, cellular BBB models enable direct measurement of endothelial inflammatory signaling, permeability, junction-associated proteins, and transcellular transport under controlled exposure conditions [36,37]. Figure 2 illustrates the structural components of the neurovascular unit and the principal mechanisms potentially contributing to SARS-CoV-2-associated BBB dysfunction.

Clinical evidence supporting the relevance of neurovascular dysfunction in SARS-CoV-2 infection has emerged from studies examining BBB integrity and associated inflammatory responses. Greene et al. (2024) characterized inflammatory, endothelial, and coagulation-related abnormalities across acute-COVID-19 severity categories [37]. In the same study, a subgroup of individuals with long COVID-19 and brain fog showed increased whole-brain BBB leakage, with regional increases in the bilateral temporal lobes and frontal cortex; BBB disruption remained detectable up to 1 year after acute infection [37]. These findings define a neurovascular phenotype in a subgroup with long-COVID-19 cognitive symptoms, although whether that BBB disruption is the initiating lesion or a universal mechanism of PASC cognitive dysfunction remains unknown.

Among participants with brain fog, circulating biomarker and PBMC transcriptomic analyses identified altered inflammatory, immune, and coagulation-related signatures. TGF-β correlated with the proportion of voxels showing abnormal leakage after correction for multiple comparisons [37]. These associations provide candidate measures for future longitudinal PASC cognitive dysfunction studies. The endothelial experiments provided functional support for this circulating neurovascular phenotype. PBMC adhesion to human brain endothelial cells was increased in long COVID-19, and serum from recovered or long-COVID-19 patients showed increased endothelial ICAM1, VCAM1, and TNF expression [37]. The combined imaging, biomarker, transcriptomic, and functional results are consistent with a neurovascular phenotype characterized by increased BBB permeability and persistent immunovascular abnormalities in a subgroup of individuals with long-COVID-19-associated cognitive symptoms.

Previous experimental studies have provided complementary mechanistic evidence for these observations. Buzhdygan et al. (2020) demonstrated that SARS-CoV-2 spike protein alters barrier function in static and microfluidic in vitro models of the human BBB [36], while Wang et al. (2024) identified BBB injury and neuroinflammatory signaling in a lung–brain microphysiological system exposed to SARS-CoV-2 [38].

By enabling controlled assessment of endothelial activation, barrier permeability, intercompartmental inflammatory signaling, pericyte signaling, matrix remodeling, transcytosis, and microvascular inflammatory responses, these models constitute a mechanistic bridge to the human findings. This convergent evidence establishes measurable variables and experimental platforms for longitudinal assessment of cognitive trajectories, biological subgrouping, and the evaluation of mechanism-matched interventions.

### 4.2. Neuroinflammation, Glial and Synaptic Mechanisms: Acute-COVID-19 and Experimental Evidence

Neurovascular signaling may connect circulating inflammatory processes to cellular responses at BBB interfaces [37,39]. Microglia and astrocytes respond to cytokine, chemokine, complement, vascular, and tissue-injury signals, thereby shaping local inflammatory programs and glial communication [22,40,41].

Activated microglia can induce a defined reactive astrocyte program, providing a cellular route through which inflammatory cues influence neural support functions [41]. In a mouse model of mild respiratory SARS-CoV-2 infection, Fernández-Castañeda et al. (2022) identified persistent white-matter-selective microglial reactivity, reduced hippocampal neurogenesis, decreased oligodendrocyte populations, and myelin loss [20]. These changes occurred alongside increased cerebrospinal-fluid (CSF) concentrations of the chemokine C-C motif chemokine ligand 11 (CCL11). Systemic administration of CCL11 reproduced hippocampal microglial reactivity and impaired neurogenesis in mice, and patients with cognitive symptoms persisting after COVID-19 had higher circulating CCL11 concentrations. Within this respiratory-infection model, CCL11 demonstrated potential as a candidate chemokine establishing a connection between peripheral inflammatory signaling with hippocampal plasticity, oligodendrocyte integrity, and myelin-related change [20].

Post-mortem human tissue provides complementary evidence from cases of severe acute COVID-19. Yang et al. analyzed single nuclei from frontal cortex and choroid plexus samples from individuals who died with severe COVID-19 and controls, identifying transcriptional perturbations in barrier and parenchymal cell populations, including interferon-, complement-, and chemokine-related programs [22]. Cell–cell communication analyses suggested altered choroid-plexus signaling to glia and neurons, and cortical T cells were detected in most of the brains from patients with COVID-19 but not the controls [22]. These severe acute-disease observations identify candidate inflammatory and barrier-associated pathways for PASC research; however, they do not demonstrate their persistence in nonfatal post-COVID-19 cognitive dysfunction.

Human brain organoids constitute a controlled model for examining the cellular consequences of direct SARS-CoV-2 exposure. Mesci et al. reported productive SARS-CoV-2 replication in induced-pluripotent-stem-cell-derived brain organoids, neural-cell death that included cortical neurons, and a loss of excitatory synapses; sofosbuvir inhibited viral replication and rescued these cellular phenotypes in the infected organoids [21]. The organoid findings defined neuron-intrinsic injury and excitatory-synapse loss as experimentally measurable outcomes, complementing the glial and barrier-associated changes identified in the mouse and post-mortem studies.

### 4.3. Oxidative Stress, Mitochondrial Mechanisms, and NAD^+^-Dependent Signaling: PASC Biomarkers and Acute/Experimental Evidence

Inflammatory activation and altered cellular metabolism can perturb redox homeostasis across endothelial, immune, glial, and neuronal compartments [42,43]. Mitochondria are an important intracellular source of reactive oxygen species (ROS) during oxidative phosphorylation. Under conditions of impaired electron transfer, mitochondrial ROS generation can increase and contribute to oxidative damage and inflammatory signaling [44]; the magnitude and direction of this response are context-dependent. Acute COVID-19 studies have reported peripheral mitochondrial DNA abnormalities that vary with infection severity [44]. Direct evidence that an oxidative mechanism persists in the CNS or causes cognitive dysfunction in long COVID-19 remains limited.

Post-COVID-19 studies have reported peripheral findings compatible with oxidative and mitochondrial disturbances, including higher serum malondialdehyde and hydroperoxides in previously infected individuals, as well as reduced platelet respiratory-chain function, oxidative phosphorylation, and endogenous coenzyme Q10 concentrations after COVID-19 [45,46]. Comparative studies of long COVID-19 and myalgic encephalomyelitis/chronic fatigue syndrome (ME/CFS) provide a basis for investigating immune–metabolic pathways in post-viral cognitive symptoms. In a pilot PBMC proteomic study, Peppercorn et al. (2023) identified overlapping immune- and mitochondria-related pathway alterations in long COVID-19 and ME/CFS [47]. However, a 7-T magnetic resonance spectroscopy study detected partly distinct brain biochemical profiles in the two conditions despite overlapping fatigue and cognitive symptoms [48]. These observations are compatible with an imbalance between mitochondrial ROS generation, antioxidant defenses, and ROS clearance that could sustain a self-amplifying redox–inflammatory cycle [49,50,51]. This pattern is consistent with a proposed ROS-amplification model in long COVID-19; however, it does not establish the presence of a generalized systemic or cerebral “ROS storm.” Current human studies have assessed circulating oxidative-damage products and antioxidant availability [45,52], as well as immune-cell bioenergetics, ROS-clearance pathways, and regional brain glutathione [49,50,51], rather than direct, cell-specific ROS flux within the CNS. Accordingly, the cellular origin, anatomical distribution, magnitude, temporal persistence, and causal relationship of ROS production with objective cognitive impairment remain unresolved. Together, these findings justify prospective long-COVID-19 cognitive cohorts that pair oxidative and mitochondrial measures with objective cognitive, neurovascular, and inflammatory outcomes.

In a human acute-COVID-19 cohort, biochemical and gene-set analyses identified increased free-radical production together with redox imbalance, and higher serum concentrations of the endothelial adhesion molecules VCAM-1 and ICAM-1 were associated with mortality [53]. Experimental studies also indicate that SARS-CoV-2 infection and selected viral proteins can disrupt mitochondrial homeostasis. In infected endothelial cells, SARS-CoV-2 increased mitochondria-derived superoxide, altered mitochondrial membrane potential, promoted mitochondrial DNA (mtDNA) release, impaired calcium responses, and activated TLR9/NF-κB (nuclear factor kappa B) signaling with cytokine release [54]. In complementary expression models, the viral proteins NSP4 and ORF9b induced mitochondrial structural injury and pro-inflammatory mtDNA release, whereas co-expression of the viroporins E and ORF3a increased mitochondrial calcium, mitochondrial ROS, cytosolic mtDNA, and NLRP3 (NOD-like receptor family pyrin domain-containing 3)-dependent IL-1β secretion [55,56].

NRF2-related antioxidant signaling represents an additional regulatory node in acute SARS-CoV-2 models. SARS-CoV-2 infection reduced NRF2 (nuclear factor erythroid 2-related factor 2) protein abundance and NRF2-dependent gene expression in human airway epithelial cells and mouse lungs, while viral NSP14 impaired SIRT1-dependent activation of the NRF2/HMOX1 (heme oxygenase 1), axis [57,58]. In endothelial cells exposed to serum from individuals with severe COVID-19, increased oxidant species and mitochondrial ROS occurred alongside reduced NRF2 nuclear accumulation and the expression of NRF2-responsive genes [59].

Izadpanah et al. examined NAD-metabolism-associated transcriptional changes following SARS-CoV-2 infection in K18-hACE2 mice and human lung datasets from lethal COVID-19 cases [42]. In the infected mouse lungs, SARS-CoV-2 induced concurrent upregulation of NAD-biosynthetic enzymes, including Naprt1, Nmnat1, Nrk, Ido1, and Nampt, and NAD-consuming enzymes such as Cd38, Parp9, Parp10, and Parp14, whereas Sirt1 expression decreased [42]. Human single-cell lung analyses further revealed cell-specific alterations, with increased NAMPT expression observed mainly in mesothelial and adventitial fibroblasts and pulmonary venous endothelial cells, while PARP14 was prominent in type I alveolar cells [42].

These findings indicate that SARS-CoV-2 infection alters NAD metabolism in a cell-dependent manner, potentially affecting the balance between NAD synthesis and consumption. Because NAD-dependent enzymes, including PARPs, CD38, and sirtuins, regulate inflammatory signaling, mitochondrial function, redox homeostasis, and cellular stress responses, disruption of this metabolic network may contribute to COVID-19 pathophysiology [42], and it makes to NAD-related pathways particularly suitable for mechanism-oriented profiling.

The available acute pulmonary, systemic, and peripheral evidence defines a focused set of neuro-COVID-19 measurements for PASC studies. These studies can quantify cell-specific NAD^+^/NADH ratios, PARP and CD38 activity, SIRT1 and SIRT3 (Sirtuin 1 and 3) expression and activity, glutathione redox balance, mitochondrial respiration, and oxidative-damage markers alongside cognitive phenotype and neurovascular biomarkers. This approach can establish how SIRT1/NF-κB, AMPK (AMP-activated protein kinase), Nrf2, and NLRP3-related processes are distributed across endothelial, glial, and neuronal compartments in post-COVID-19 cognitive dysfunction. However, is important to note that changes in messenger-RNA abundance should be interpreted as transcriptional observations only; they do not demonstrate enzyme activity, metabolite flux, or cellular NAD^+^ availability without corresponding functional measurements.

### 4.4. Plasticity, Neurogenesis, and miRNA Regulation: Experimental Evidence and PASC Biomarker Hypotheses

Hippocampal neurogenesis and synaptic plasticity provide cellular outcomes through which inflammatory, neurovascular, and metabolic signaling may be related to memory, learning, and executive function after COVID-19. These processes offer a focused opportunity to connect the glial and redox pathways discussed above and the cognitive domains that define the clinical phenotype. In this sense, Soung et al. (2022) examined the hippocampal consequences of acute SARS-CoV-2 infection in hamsters and in hippocampal tissue from people who died with acute COVID-19 [60]. Their results identified microglial activation and increased IL-1β and IL-6 expression, particularly in the hippocampus and medulla oblongata. Both hamster and human tissue also showed fewer neuroblasts and immature neurons in the dentate gyrus. In the hamster model, abnormal BBB permeability accompanied the hippocampal phenotype in the absence of viral neuroinvasion, highlighting inflammatory and neurovascular signaling as experimentally supported routes to altered hippocampal plasticity [60]. This acute-infection and post-mortem evidence defines hippocampal cytokine expression, microglial activation, dentate-gyrus neuroblasts, and immature neurons as measurable endpoints for longitudinal PASC studies and can be integrated with neuropsychological measures of memory and executive function, neurovascular biomarkers, and imaging markers of hippocampal structure or connectivity.

The SIRT1–miR-134-CREB (cAMP response element-binding protein)/BDNF (brain-derived neurotrophic factor) pathway provides an experimentally defined molecular association between cellular metabolic regulation and synaptic plasticity. Gao et al. (2010) showed that SIRT1 activation enhanced synaptic plasticity and memory through a miR-134-dependent mechanism, whereas reduced SIRT1 activity was associated with higher miR-134 expression and lower CREB and BDNF expression [61]. The study identified a YY1 (Yin Yang 1 transcription factor)-containing repressor complex through which SIRT1 regulates miR-134 transcription, thereby linking metabolic state to CREB/BDNF-dependent synaptic function. Baby et al. (2020) extended the plasticity relevance of this regulatory axis in an Aβ1–42 (Amyloid beta peptide 1–42) hippocampal model of AD [62]. In that model, miR-134-5p was increased, and its inhibition restored late long-term potentiation and synaptic tagging/capture while increasing CREB-1 (cAMP response element-binding protein 1) and BDNF expression. The findings of these non-COVID-19 experimental studies establish the SIRT1–miR-134–CREB/BDNF axis as a mechanistically coherent pathway for studying the relation among metabolic regulation, synaptic plasticity, and memory-related outcomes.

Human PASC miRNA data may add a peripheral biomarker dimension to this experimental research. Carreras-Badosa et al. (2026) profiled plasma miRNA in 48 people with post-COVID-19 condition, classified according to neuropsychological testing, and 16 controls [63]. Thirteen miRNAs differed between cognitively impaired and cognitively preserved post-COVID-19 groups, and several validated miRNAs correlated with memory, processing-speed, and executive-function measures. The reported plasma signature, including miR-369-3p, miR-448, miR-450a-5p, and miR-483-3p, provides a clinical biomarker set that is distinct from the miR-134 pathway studied in experimental plasticity models. This distinction creates a tractable translational program and a research area of opportunity. Well-phenotyped PASC cohorts can relate serial peripheral miRNA profiles to cognitive performance, neurovascular and inflammatory markers, and, where feasible, CSF or imaging measures of hippocampal integrity. Human-relevant neural and neurovascular systems can then examine how participant-derived inflammatory or endothelial signals influence miRNA regulation, CREB, BDNF, dendritic structure, synaptic function, and neurogenesis.

The following section examines the anatomical and physiological routes through which SARS-CoV-2-associated signals may reach or influence these neural and neurovascular compartments.

### 4.5. Evidence–Weighted Routes Through Which Acute SARS-CoV-2-Associated Signals May Contribute to Post-COVID-19 Brain Dysfunction

SARS-CoV-2-associated signals can influence the CNS through several interconnected routes: the olfactory mucosa–CNS interface, the hematogenous and neurovascular circulation, the choroid plexus–blood–cerebrospinal fluid barrier, and systemic organ-to-brain signaling. Evidence for these routes comes predominantly from acute infection, autopsy material, and experimental systems; their contribution to persistent cognitive dysfunction is likely to vary with disease severity, host factors, timing after infection, and the tissue compartment examined.

The olfactory mucosa represents a unique interface where epithelial, vascular, immune, and neural components converge. In autopsy tissue from patients with acute COVID-19, Meinhardt et al. (2021) detected SARS-CoV-2 RNA and protein across the nasopharynx and brain, finding a distribution extending from the olfactory mucosa to neuroanatomical regions including the medulla oblongata [64]. These findings position the olfactory interface as a relevant site for examining the interplay among local infection, barrier disruption, inflammatory signaling, and CNS involvement. In PASC, integrating longitudinal olfactory assessment with nasal or mucosal biomarkers, neuroimaging, and cognitive phenotyping could help define the contribution of these processes across clinical subgroups.

The hematogenous and neurovascular route links systemic inflammatory, thromboinflammatory, and hypoperfusion-related processes with the cerebral microvasculature [37,65]. Circulating viral proteins, inflammatory mediators, coagulation products, and other serum factors can engage the vascular endothelium and modulate BBB integrity, providing a mechanistic interface between peripheral alterations and CNS dysfunction. In a subgroup with long-COVID-19 cognitive impairment, Greene et al. (2024) identified this neurovascular phenotype by integrating dynamic contrast-enhanced MRI evidence of BBB leakage with peripheral immune and coagulation signatures, enhanced PBMC adhesion to human brain endothelial cells, and inflammatory endothelial responses to participant serum [37].

The choroid plexus represents a distinct CNS barrier interface in which specialized epithelial cells regulate blood–CSF exchange and contribute to the molecular and immune composition of the CSF. Using human pluripotent stem cell-derived brain and choroid plexus organoids, Pellegrini et al. (2020) identified ACE2 expression predominantly in mature, lipoprotein-producing choroid plexus epithelial cells, together with expression of SARS-CoV-2 entry-associated proteases [66]. Exposure to spike pseudovirus and live SARS-CoV-2 revealed preferential viral tropism for these epithelial cells, whereas neurons and glial cells showed little to no infection in this experimental system. Infection disrupted the integrity of the choroid plexus epithelium and increased barrier permeability, affecting an interface that normally restricts the passage of pathogens, immune cells, and inflammatory mediators into the CSF. These findings identify the blood–CSF barrier as a biologically distinct site of SARS-CoV-2 susceptibility and provide an experimental basis for investigating how epithelial injury can alter communication between the systemic circulation, CSF, and CNS. Choroid plexus organoids therefore offer a tractable platform for examining infection-induced changes in barrier function and CSF-facing inflammatory and metabolic signaling, complementing studies focused on the cerebral microvascular BBB.

Finally, systemic organ-to-brain signaling provides a fourth route through which pulmonary inflammation, hypoxemia, vascular and coagulation-related disturbances, autonomic symptoms, gastrointestinal alterations, and metabolic stress may shape CNS homeostasis. In a prospective cohort of survivors of severe COVID-19 requiring intensive care, sustained systemic inflammatory markers during admission were associated with higher plasma neurofilament-light concentrations and with cognitive impairment six months after discharge [67]. Evidence from Greene et al. (2024) further linked peripheral inflammatory and coagulation-related abnormalities with BBB disruption and cognitive symptoms in a long-COVID-19 subgroup [37]. Intestinal barrier disruption, microbial translocation, and persistent alterations in gut microbial composition may provide an indirect systemic route through which acute SARS-CoV-2 infection influences the CNS [68]. In severe acute COVID-19, markers of impaired gut-barrier integrity and microbial translocation were associated with systemic inflammation and immune activation [68]. Post-COVID-19 studies have also reported persistent intestinal dysbiosis and, in patients with inflammatory bowel disease, intestinal SARS-CoV-2 antigen persistence months after infection [69,70].

The four routes by which acute SARS-CoV-2 infection, or its systemic consequences, may influence the CNS provide an evidence-weighted anatomical and physiological background for the candidate mechanisms described in Section 4.1, Section 4.2, Section 4.3 and Section 4.4. Figure 3 summarizes the four routes and their convergence on neurovasular, immune, metabolic, and network-level processes relevant to post-COVID-19 cognitive dysfunction.

### 4.6. Neuroanatomical and Functional-Network Alterations in Post-COVID-19 Cognitive Dysfunction

Post-COVID-19 cognitive symptoms arise in functions supported by distributed neural systems. Attention, executive control, processing speed, working memory, and episodic memory depend on coordinated interactions among the prefrontal, parietal, thalamic, basal ganglia, temporal, and hippocampal regions [71,72]. The hippocampal formation is especially relevant to episodic memory and contextual learning, whereas frontoparietal systems support executive control and sustained attention. This distributed organization provides a neuroanatomical basis for the heterogeneous cognitive profiles described in post-COVID-19 cohorts. Dacosta-Aguayo et al. (2024) combined neuropsychological testing with diffusion and functional MRI in 53 people assessed approximately 1.8 years after acute SARS-CoV-2 infection [73]. Executive, attentional, and memory deficits were identified in 59%, 55%, and 40% of participants, respectively. Higher frontoparietal resting-state activity was associated with lower memory performance, and diffusion measures in white-matter tracts also correlated with memory. The study further identified hippocampal–amygdala and hippocampal–temporal connectivity differences; these latter associations did not remain significant after false-discovery-rate correction [73]. The importance of this study is that it identified network activity and white-matter microstructure as measurable correlates of cognition in a defined long-COVID-19 cohort.

Acute infection studies supply complementary evidence of hippocampal vulnerability. Soung et al. (2022) identified increased IL-1β and IL-6 expression, microglial activation, and fewer neuroblasts and immature neurons in the dentate gyrus of SARS-CoV-2-infected hamsters and in hippocampal tissue from people who died with acute COVID-19 [60]. The dentate gyrus contains the adult subgranular-zone neurogenic niche; thus, these findings support a connection between acute inflammatory signaling and cellular processes relevant to hippocampal plasticity. Authors also defined hippocampal cytokine activity, microglial state, neuroblast abundance, and immature-neuron markers as candidate outcomes for human-relevant experimental systems [60].

Adult hippocampal neurogenesis includes the proliferation, maturation, and integration of neural progenitors within the dentate-gyrus subgranular zone [74]. Angiogenesis refers to the formation and remodeling of microvessels. Palmer et al. (2000) showed that dividing cells in the adult dentate gyrus cluster around vasculature and that approximately 37% express endothelial markers, identifying an angiogenic niche in which local blood vessels and cellular plasticity were closely associated [75]. Synaptic plasticity encompassed activity-dependent changes in synaptic strength, receptor trafficking, dendritic-spine organization, and circuit connectivity. These distinctions allow each process to be linked to an appropriate endpoint and considered as a distinct biological mechanism contributing to brain fog. In the literature on non-COVID-19 neurological and viral disease, activated microglia and astrocytes release cytokines, chemokines, and nitric oxide, with accompanying changes in synaptic proteins such as synaptophysin and drebrin [76].

The acute-infection hippocampal findings identify neuroblast abundance, immature-neuron markers, hippocampal cytokine activity, and microglial state as experimental-model outcomes. They should not be presented as directly quantifiable endpoints in living PASC participants. In longitudinal PASC studies, clinically feasible correlates include objective cognitive assessment, structural and functional MRI, and prospectively defined peripheral or CSF biomarkers where appropriate; neuroblast abundance and immature-neuron measures remain reserved for experimental or post-mortem systems.

## 5. Resveratrol: Pharmacology and Translational Constraints

Resveratrol is a polyphenolic stilbene with activity across inflammatory, redox, metabolic, endothelial, and cellular-stress pathways in experimental systems [4,10,11] (Figure 4). Its translational relevance to post-COVID-19 cognitive dysfunction depends on the alignment among clinically achievable exposure of parent resveratrol or active metabolites, modulation of a prespecified biological target in the relevant tissue compartment, and improvement in a validated patient-relevant outcome.

### 5.1. Absorption, Metabolism, Central Nervous System Exposure, and Safety

Orally administered resveratrol is absorbed and undergoes rapid pre-systemic metabolism. In six healthy volunteers receiving 25 mg of radiolabeled resveratrol, Walle et al. (2004) estimated absorption of at least 70%; unchanged parent resveratrol remained below 5 ng/mL in plasma [80]. Sulfate and glucuronide conjugation in the intestine and liver accounted for much of this exposure profile. These data make parent compound, sulfate and glucuronide metabolites, dose, formulation, sampling time, and biological matrix essential variables in translational studies.

Resveratrol CNS exposure has been examined in other non-PASC clinical populations. Turner et al. (2015) conducted a 52-week randomized, placebo-controlled phase 2 trial in 119 participants with mild-to-moderate Alzheimer’s disease (AD) [81]. Treatment escalated from 500 mg once daily to 1000 mg twice daily; parent resveratrol and major metabolites were measurable in plasma and CSF. Nausea, diarrhea, and weight loss were the most frequent adverse events, and MRI showed greater brain-volume loss in the resveratrol group. This study informed formulation-specific exposure, CSF sampling, tolerability surveillance, and interpretation of imaging measures during high-dose treatment [81]. The translational objective for post-COVID-19 cognitive dysfunction will be therefore to connect pharmacokinetics with prespecified biological and clinical measures in the intended population.

Nominal concentrations used in cellular experiments should not be equated with human plasma or CSF concentrations: a valid exposure comparison requires dose, formulation, sampling time, free parent compound, and conjugated-metabolite measurements. Accordingly, the experimental literature identifies pharmacodynamic hypotheses but does not establish that matched target-site exposures are achievable in PASC.

### 5.2. Experimental Evidence from Non-COVID-19 Neurological Models: Relevance and Limits for PASC

Neurodegenerative diseases are characterized by progressive neuronal dysfunction and loss [82]. Resveratrol has shown neuroprotective potential in models of AD [83,84], Parkinson’s disease [85], amyotrophic lateral sclerosis (ALS) [86], Huntington’s disease, and vascular dementia [87]. These effects are partly associated with activation of SIRT1, a NAD^+^-dependent deacetylase involved in oxidative stress (OS), mitochondrial function, neuronal survival, and regulation of NF-κB-mediated neuroinflammation [81,87]. Table 2 summarizes the reported effects of resveratrol across these neurodegenerative disorders.

AD is characterized by progressive cognitive decline and the accumulation of β-amyloid (Aβ) peptides and hyperphosphorylated tau, which contribute to synaptic dysfunction, OS, neuroinflammation, and neuronal degeneration [94]. IFITM3 may further promote amyloidogenesis by interacting with the γ-secretase complex and increasing Aβ production [95]. Because IFITM3 expression is also increased during SARS-CoV-2 infection, this mechanism provides a potential link between antiviral immune responses and amyloidogenic pathways [96]. In Tg2576 mice, Wang et al. (2006) reported that Cabernet Sauvignon attenuated spatial memory deterioration and Aβ neuropathology, with evidence of increased non-amyloidogenic APP processing [83]. These findings cannot be attributed specifically to resveratrol because the intervention consisted of whole wine. In SAMP8 mice, dietary resveratrol reduced cognitive impairment, amyloid burden, and tau hyperphosphorylation [84]. In a 52-week phase II randomized controlled trial involving 119 patients with mild-to-moderate AD, resveratrol doses were escalated to 2 g/day. Resveratrol and its major metabolites were detected in CSF, and treatment attenuated the decline in CSF Aβ40 concentrations compared with placebo [81]. A retrospective analysis of samples from 38 participants in the same trial found reduced CSF MMP-9 and increased CSF IL-4, macrophage-derived chemokine, and FGF-2 concentrations compared with placebo [88]. These biomarker findings do not establish clinical efficacy.

In Parkinson’s disease, characterized by dopaminergic neuronal loss associated with mitochondrial dysfunction, OS, and neuroinflammation, resveratrol protected SH-SY5Y cells against rotenone-induced toxicity by reducing OS, suppressing COX and NADH oxidase and activating the SIRT1 and AMPK signaling pathways [85]. Similarly, in ALS models, resveratrol delayed disease onset, preserved motor function, and prolonged survival, with effects associated with SIRT1/AMPK signaling, autophagic flux, and mitochondrial biogenesis [86].

In Huntington’s disease models, resveratrol restored PGC-1α and TFAM protein levels and mitochondrial DNA content in patient-derived lymphoblasts and improved motor coordination and motor learning in YAC128 mice [90]. In contrast, Ho et al. (2010) reported that the resveratrol formulation SRT501-M increased PGC-1α and downstream gene expression in brown adipose tissue but did not significantly improve motor performance, survival, or striatal atrophy in N171-82Q mice [89].

In mice with chronic EAE, resveratrol attenuated retinal ganglion cell loss without significantly suppressing inflammation during the chronic phase examined. In experimental optic neuritis, intravitreal SRT501 (a resveratrol formulation), attenuated retinal ganglion cell loss without preventing optic nerve inflammation. Sirtinol blocked this neuroprotective effect, supporting SIRT1 involvement [91,92].

Across non-COVID-19 neurological models, reported neuroprotective actions of resveratrol converge on the modulation of neuroinflammation, OS, mitochondrial function, and cellular survival pathways. Its effects on endothelial function and cerebral blood flow may provide additional mechanisms contributing to neuronal and cognitive protection [81,83,84,86,87,91,92]. However, those effects should be interpreted as indirect evidence that informs target selection, and the relevance of a given pathway must be tested in a post-COVID-19 phenotype with objective cognitive outcomes and formulation-specific exposure measurements.

### 5.3. Biological Actions of Resveratrol Relevant to Neuro-COVID-19

BBB biology is especially relevant to resveratrol because a CNS-oriented intervention can be evaluated across three related characteristics: exposure at the neurovascular or parenchymal site of action; modulation of a prespecified barrier, inflammatory, or metabolic target; and a change in a patient-relevant clinical outcome. Preclinical studies of resveratrol in non-COVID-19 endothelial or barrier-injury models can inform target selection and pharmacodynamic assays. A PASC cognitive impairment study can build from these data by measuring parent resveratrol and relevant metabolites, neurovascular target engagement, safety, and objective cognition within the same protocol. Figure 5 identifies the preclinical resveratrol actions that can be carried forward as testable pharmacodynamic hypotheses.

Experimental studies have evaluated endothelial signaling role as a central domain of resveratrol action (Figure 4 and Figure 5). In cultured coronary arterial endothelial cells, resveratrol increased Nrf2 transcriptional activity and expression of the Nrf2-responsive genes NQO1 (NAD(P)H quinone dehydrogenase 1), GCLC (glutamate-cysteine ligase catalytic subunit), and HMOX1 while reducing high-glucose-induced mitochondrial and cellular oxidant stress. In high-fat-diet-fed mice, the same study associated resveratrol with lower vascular oxidant stress, improved acetylcholine-mediated vasodilation, and reduced vascular apoptosis; these effects were substantially attenuated in Nrf2-deficient animals [77]. This study supports the use of Nrf2-responsive genes, vascular OS, and endothelial function as measurable pharmacodynamic outcomes.

SIRT1- and AMPK-associated signaling provides a second endothelial route. Gracia-Sancho et al. (2010) showed that resveratrol-induced SIRT1 activation increased KLF2 expression through a MEK5 (mitogen-activated protein kinase kinase 5)/MEF2 (myocyte enhancer factor 2)-dependent mechanism in human vascular endothelial cells [78]. In TNF-α-stimulated human umbilical-vein endothelial cells (HUVECs), Chen et al. (2013) reported reduced inflammatory-factor expression and increased autophagy after resveratrol treatment [4]. Pharmacological inhibition and siRNA (Small interfering RNA) experiments identified cAMP, protein kinase A, AMPK, SIRT1, ATG5 (Autophagy-related protein 5), and BECN1 (Beclin 1) as components of the response in that model. Resveratrol also modifies mitochondrial redox responses in an oxidant-injury model. In tert-butyl-hydroperoxide-challenged HUVECs, Zhou et al. (2014) found preserved mitochondrial membrane potential, lower mitochondrial ROS and apoptosis, and higher SOD2 (superoxide dismutase 2), glutathione-peroxidase, and IDH2 (isocitrate dehydrogenase 2) activity after resveratrol exposure [79]. AMPK inhibition and knockdown of AMPK, PGC-1α, or SIRT3 altered these responses, identifying an AMPK–PGC-1α–ERRα–SIRT3-associated mechanism within that experimental setting [79].

In the N9 murine microglial cell line stimulated with lipopolysaccharide and ATP, Tufekci et al. (2021) reported that resveratrol reduced NLRP3 inflammasome signaling, pro-inflammatory cytokine production, pyroptotic cell death, NF-κB activity, and miR-155 expression while promoting AMPK/SIRT1 activation [97]. These findings added an inflammatory-cell perspective and provided a molecular basis for evaluating the relevance of these pathways in human glial and neurovascular systems.

The antiviral study by Pasquereau et al. (2021) screened seven candidate compounds in cell-culture assays of human coronavirus 229E (HCoV-229E) [11]. Resveratrol reduced the viral titer by approximately 80% at an estimated half-maximal effective concentration of 4.6 µM and showed limited cytotoxicity in that system. After this initial screening, resveratrol was also the only active candidate among resveratrol, lopinavir/ritonavir, and chloroquine to reduce the SARS-CoV-2 viral titer with reduced cytotoxicity [11]. In the same sense, ter Ellen et al. (2021) tested both resveratrol and pterostilbene in SARS-CoV-2-infected African green monkey kidney epithelial cells (Vero cells) and in differentiated primary human bronchial epithelial cells cultured at an air–liquid interface [98]. In post-inoculation experiments, both compounds reduced the production of infectious viral progeny, indicating an effect on intracellular replication rather than exclusively on viral entry. In the primary airway–epithelium model, antiviral activity was still observed up to 48 h after infection without replenishment of the compounds [98]. As these experiments have established assay systems for acute antiviral hypotheses, the post-COVID-19 cognitive question centers on exposure, target engagement, neurovascular biology, and cognitive outcome after the acute infectious phase.

## 6. Candidate Biological Targets for Resveratrol in Post-COVID-19 Cognitive Dysfunction

The preceding sections identified biological processes relevant to post-COVID-19 cognitive dysfunction, including neurovascular inflammation and BBB permeability, altered immune and coagulation signaling, glial inflammatory responses, oxidative and mitochondrial stress, and, during acute infection, viral replication. Resveratrol has shown measurable effects in endothelial, microglial, and airway-cell models, providing testable links between PASC-related processes and potential therapeutic targets. The neurovascular domain offers the strongest connection, as reported by Greene et al. (2024) [37]. In their study, BBB permeability, circulating inflammatory and coagulation abnormalities, PBMC adhesion to brain endothelial cells, and serum-induced endothelial inflammatory responses were increased in participants with long-COVID-19 cognitive symptoms [37]. In the same sense, in a separate non-COVID-19 endothelial model, resveratrol reduced TNF-α-induced inflammatory-factor expression through a cAMP–PKA–AMPK–SIRT1-associated autophagy pathway [4]. This pairing identifies a practical early-phase study approach: enroll a clinically characterized neurovascular subgroup, document parent-compound and metabolite exposure, measure endothelial and inflammatory variables, include neurovascular imaging where feasible, and assess objective cognition and everyday function [4]. Table 3 presents the crosswalk by candidate domain, directness of the PASC evidence, primary resveratrol evidence, and the next experiment required. This table also distinguishes clinical associations, models, and pharmacodynamic or clinical endpoints, establishing an evidence-based sequence from biological observations to target engagement and patient-relevant outcomes.

The redox, mitochondrial, and NAD^+^-related domains provide a second set of candidate measurements, since previous studies report coordinated changes in NAD-biosynthetic and NAD-consuming gene expression during acute SARS-CoV-2 infection in pulmonary models and datasets [42]. Along the same line, Zhou et al. (2014) showed that resveratrol modified mitochondrial ROS, membrane potential, apoptosis, and antioxidant-enzyme activity in oxidant-challenged endothelial cells through AMPK–PGC-1α–ERRα–SIRT3-associated signaling [79]. Together, these studies support a translational program that measures redox state, NAD^+^-related enzymes, mitochondrial function, parent compound and metabolite exposure, and its pre-specified relation to cognition.

Inflammasome-related evidence provides a complementary cellular hypothesis, as observed by Soung et al. (2022), who reported hippocampal cytokine expression, microglial activation, and reduced neurogenesis during acute COVID-19 in hamster and post-mortem human tissue [60]. Tufekci et al.’s study using the lipopolysaccharide (LPS)/ATP-stimulated N9 microglial cell line, they found that resveratrol reduced NLRP3 and NF-κB signaling, cytokine production, pyroptosis, and miR-155 expression [97]. These findings support testing these mechanisms in human-relevant glial and neurovascular models and selecting corresponding exploratory biomarkers for clinical studies.

Across these mechanisms, objective cognition provides the common patient-relevant anchor, while adequate resveratrol exposure is essential for linking target engagement to clinical effects. Considering that resveratrol and its major metabolites are measurable in plasma and CSF in AD, providing information on exposure and tolerability [81], a PASC trial could integrate a standardized formulation and longitudinal pharmacokinetic sampling with a validated cognitive composite, measures of everyday function, and mechanism-matched secondary outcomes.

## 7. Resveratrol: Translational Human Evidence and Clinical Implications

The human evidence relevant to resveratrol and post-COVID-19 cognitive dysfunction may be divided considering four distinct studies: short-term use during acute COVID-19, exposure and tolerability in non-COVID-19 populations, cognitive and cerebrovascular outcomes outside PASC, and the design of an early-phase study in the intended clinical population.

### 7.1. Human Studies of Resveratrol’s Usefulness for the Establishment of a PASC Cognitive Trial

Acute-COVID-19 trials provide information on short-term tolerability and peripheral outcomes. In a phase 2 outpatient trial by McCreary et al. (2022), 100 evaluable participants received resveratrol or placebo [99]. Hospitalization occurred in 2% and 6% of participants, emergency-department visits in 8% and 14%, and pneumonia in 8% and 16%, respectively. Event estimates had wide confidence intervals, and the investigators characterized the study as underpowered because of the small sample and low event rate. No clinically significant adverse event was attributed to resveratrol [99].

Bastin et al. (2025) randomized 44 participants with acute COVID-19 to resveratrol at 750 mg/day or placebo for 10 days [100]. The resveratrol group showed lower concentrations of C-reactive protein (CRP), IL-1β, TNF-α, fasting glucose, white blood cells, platelets, and neutrophils, together with higher lymphocyte counts. This study contributes short-duration evidence on peripheral inflammatory and hematological measures during acute infection.

Studies in non-PASC populations inform outcome selection for a cognitive trial but show mixed results. Witte et al. (2014) randomized 46 healthy overweight adults aged 50–75 years to 200 mg/day of resveratrol plus 320 mg/day quercetin or placebo for 26 weeks and reported improved 30 min word retention, increased hippocampal functional connectivity, and changes in HbA1c and body fat [101]. In a separate 26-week randomized trial in 42 participants with mild cognitive impairment, of whom 40 completed the intervention (200 mg/d resveratrol combined with 350 mg/d quercetin, *n* = 18; or placebo: 1015 mg/d olive oil, n = 22; n = 30 in the longitudinal fMRI subsample), Köbe et al. (2017) found no significant between-group difference in primary declarative memory performance (Auditory Verbal Learning Test, AVLT), despite increased resting-state functional connectivity between the left hippocampus and prefrontal/parietal regions alongside reduced glycated hemoglobin (HbA1c) [102]. Because both Witte and Köbe evaluated combined formulations incorporating quercetin (added to enhance bioavailability), the biological, neuroimaging, and behavioral outcomes from these trials cannot be attributed exclusively to resveratrol. This combined administration represents a key methodological confounder that limits the direct extrapolation of these findings to single-agent resveratrol interventions in PASC [101,102]. In 80 postmenopausal women, Evans et al. (2017) reported increased cerebrovascular responsiveness with improvements in verbal memory and overall cognitive performance after 14 weeks of treatment [103]. In a longer 24-month crossover trial in 125 postmenopausal women, Thaung Zaw et al. (2021) reported a 33% improvement in overall cognitive performance, defined as a composite performance score across a battery covering verbal memory, executive function, and spatial processing-compared to placebo [104]. This cognitive benefit was closely associated with a 17% increase in cerebrovascular responsiveness to hypercapnia and improved cerebral blood flow velocity, supporting microvascular reactivity as a key mediator of cognitive maintenance in aging cohorts [104]. However, biological modulation does not always translate into detectable cognitive gains.

The pharmacokinetic and CSF findings from Turner et al. (2015), discussed in Section 5.1, add formulation-specific information on high-dose exposure and tolerability in AD [81]. The acute-COVID-19, aging, menopause, and AD studies identify a set of design variables, dose, formulation, parent compound and metabolite sampling, safety surveillance, cognitive testing, and vascular measures that can be adapted for PASC evaluation. Table 4 summarizes these studies by population, intervention, outcomes, and direct relevance to the PASC cognitive question.

### 7.2. Current Management of Post-COVID-19 Cognitive Dysfunction and the Role of Resveratrol

Clinical management of post-COVID-19 cognitive dysfunction begins with characterization of the cognitive complaint and identification of contributors amenable to established care. The multidisciplinary guidance by Fine et al. (2022) supports assessment of sleep, mood, fatigue, autonomic symptoms, cardiopulmonary limitations, medication effects, and metabolic or endocrine conditions alongside cognitive and functional evaluation [105]. Cognitive rehabilitation can address functional goals through compensatory strategies, restorative training, pacing, return-to-work support, and treatment of coexisting symptoms.

A 12-week pilot randomized trial of an adapted cognitive-rehabilitation intervention reported improved self-reported cognition in participants with objective post-COVID-19 impairment; objective-domain outcomes provide a useful basis for larger confirmatory trials [106]. In a separate randomized trial of 40 people with long COVID-19, individualized cardiopulmonary rehabilitation improved perceived stress and depression, while neuropsychological test performance remained similar between the intervention and control groups [107]. These trials illustrate the value of aligning the clinical claim with the outcome that changed.

Pharmacological approaches for post-COVID-19 cognitive symptoms remain exploratory and can be used to address defined conventional indications under appropriate clinical supervision. Case series of methylphenidate describe symptomatic treatment experiences and provide candidate questions on patient selection, dose, duration, and safety for future controlled studies [108,109].

Within this clinical landscape, resveratrol occupies a research-adjunct position, with its potential contribution evaluated alongside standard diagnostic assessment, rehabilitation, and treatment of coexisting disorders. Table 5 summarizes current management options and the research role of resveratrol.

## 8. Concluding Remarks and Future Directions

PASC cognitive dysfunction should not be viewed as the consequence of a single residual lesion or a uniform inflammatory state. Available human and experimental evidence instead support partially overlapping neurovascular, immune, metabolic, and systemic routes that may converge on CNS homeostasis. In cohorts with cognitive symptoms or severe acute disease, BBB disruption, altered immunovascular interactions, persistent systemic inflammation, and neuroaxonal-injury biomarkers have been associated with subsequent cognitive abnormalities. At the molecular level, endothelial-junction remodeling, glycocalyx disruption, and leukocyte–endothelial adhesion provide plausible routes by which circulating inflammation can alter barrier function. Complementary experimental studies show that SARS-CoV-2 infection or expression of selected viral proteins can increase mitochondrial ROS, alter calcium handling and membrane potential, release mtDNA, and activate TLR9/NF-κB or NLRP3-dependent inflammatory signaling. Reduced NRF2-related antioxidant signaling in infected epithelial cells and inflamed endothelial models may further impair redox control. These mechanisms provide biologically plausible explanations linking resveratrol-responsive pathways with processes implicated in PASC cognitive dysfunction. Resveratrol should therefore be regarded as an investigational multi-node candidate, not an evidence-based treatment.

Future studies should prioritize biomarker-enriched randomized controlled trials in people with objectively characterized post-COVID-19 cognitive dysfunction. Enrollment criteria should define the predominant cognitive phenotype and document relevant confounders, including demographic characteristics, premorbid conditions, sleep, mood, fatigue, autonomic symptoms, and acute-illness exposures. Trials should use chemically characterized resveratrol formulations, matched placebo, multidisciplinary usual care, longitudinal pharmacokinetic sampling of parent compound and relevant metabolites, and validated cognitive composites aligned with the predominant impairment. Secondary outcomes should include everyday function, patient-reported cognition, fatigue, sleep, mood, safety, and prospectively selected neurovascular, inflammatory, or redox measures where feasible. Figure 6 visualizes this integrated workflow spanning objective phenotyping through randomized evaluation phases emphasizing iterative feedback loops refining mechanistic understanding endpoint relevance and clinical applicability.

Safety monitoring remains essential and should include systematic assessment of gastrointestinal tolerability, liver enzyme levels, concomitant medication use, and potential drug–drug interactions, particularly in patients with post-viral conditions who are receiving multiple medications. Mechanistic analyses within these trials could help determine whether changes in circulating inflammatory and endothelial biomarkers, improvements in redox homeostasis, and, where feasible, neurovascular alterations assessed through BBB imaging or validated measures are associated with improvements in cognitive outcomes. By combining methodological rigor with population-specific adaptations, future trials may strengthen the clinical validation of therapeutic strategies across different etiologies of cognitive dysfunction. Such approaches could help bridge the gap between preclinical findings and clinical implementation while ensuring patient safety and generating robust evidence to support regulatory decisions and clinical practice. Mediation analyses can help explore potential causal mechanisms, but they cannot independently establish causal relationships. Causal interpretation requires an appropriate study design, clear temporal ordering of measurements, adequate control of confounding, and consideration of the assumptions underlying mediation analysis.

## Figures and Tables

**Figure 1:**
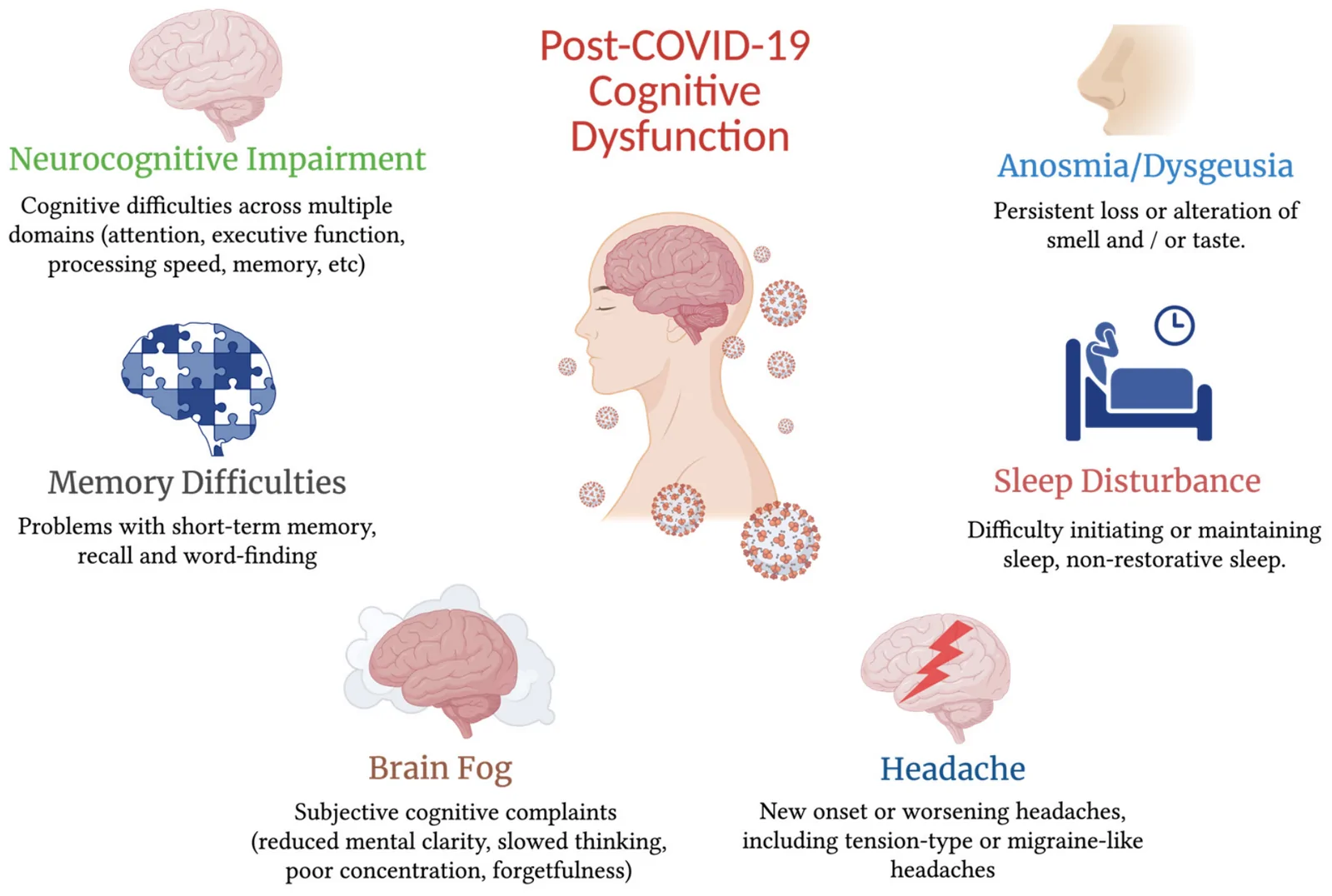
Neurological and cognitive manifestations after COVID-19. Post-COVID cognitive dysfunction is heterogeneous phenotype and not a single diagnostic entity. Subjective “brain fog” may include reduced mental clarity, slowed thinking, forgetfulness, and difficulty sustaining attention; these complaints can coexist with fatigue, sleep disturbance, headache, and affective symptoms. Across studies, objective deficits have most often involved attention, executive function, processing speed, memory, and working memory, although affected domains and effect sizes vary with cohort selection, acute illness severity, follow-up interval, assessment method, vaccination, reinfection, and comorbidity [2,3].

**Figure 2:**
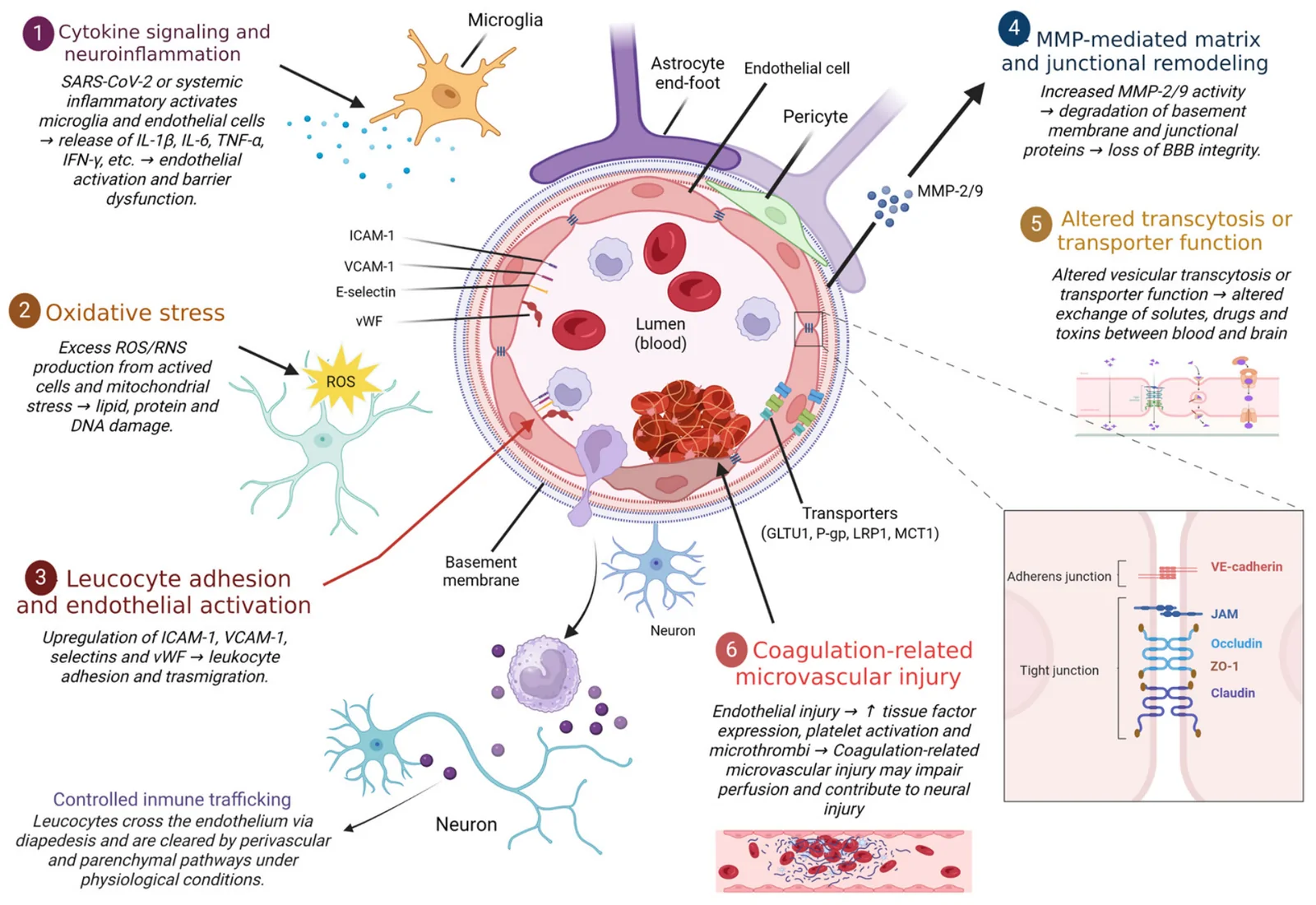
Neurovascular unit and candidate mechanisms of SARS-CoV-2-associated injury. The neurovascular unit comprises brain endothelial cells linked by tight and adherens junctions, basement membrane, pericytes, astrocyte end-feet, microglia, neurons, transport systems, and regulated immune-cell trafficking. Candidate SARS-CoV-2 injury mechanisms include cytokine-mediated endothelial and glial activation (1), oxidative stress (2), leukocyte adhesion (3), matrix remodeling (4), altered transcytosis or transporter function (5), and coagulation-related microvascular injury (6). The numbered mechanisms represent potential sites and processes of SARS-CoV-2-associated neurovascular injury, supported by varying levels of experimental and clinical evidence. The figure include the neurovascular components potentially involved and do not necessarily imply direct causal relationships. Human long-COVID-19 data support BBB leakage and systemic inflammatory/coagulation abnormalities in a cognitively impaired subgroup [37]. SARS-CoV-2 or spike exposure can alter barrier properties and induce endothelial inflammatory responses [36,38]. The model does not require persistent productive neuronal infection. BBB, blood–brain barrier; GLUT1, glucose transporter 1; ICAM-1, intercellular adhesion molecule 1; IFN-γ, interferon gamma; IL-1β, interleukin-1 beta; IL-6, interleukin 6; LRP1, low-density lipoprotein receptor-related protein 1; MCT1 monocarboxylate transporter 1; MMP-2/9, matrix metalloproteinases 2 and 9; P-gp, P-glycoprotein; RNS, reactive nitrogen species; ROS, reactive oxygen species; TNF-α, tumor necrosis factor alpha; VCAM-1, vascular cell adhesion molecule 1; VE-cadherin, vascular endothelial cadherin; vWF, von Willebrand factor; ZO-1, zonula occludens 1.

**Figure 3:**
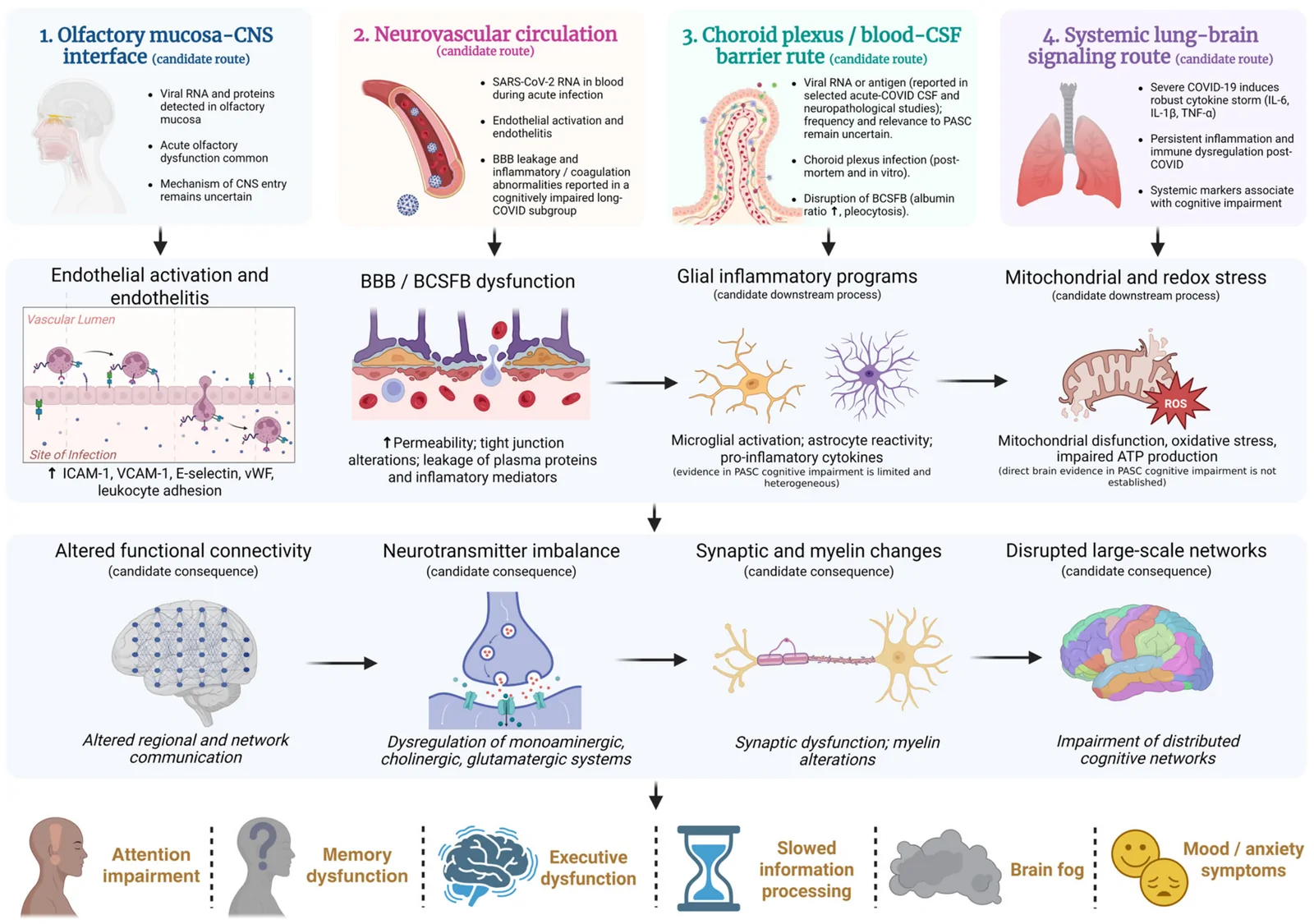
Evidence-weighted model of candidate pathways contributing to post-COVID-19 cognitive dysfunction. The schematic depicts four nonexclusive routes by which acute SARS-CoV-2 infection, or its systemic consequences, may influence the CNS: the olfactory mucosa–CNS interface, neurovascular circulation, the choroid plexus/blood–cerebrospinal fluid barrier, and systemic lung–brain signaling. Human evidence supports an association between long-COVID-19 cognitive impairment and BBB leakage, systemic inflammation, coagulation-related signatures, and endothelial activation in a defined subgroup [37]. Acute-COVID-19 autopsy and experimental studies support olfactory-mucosal viral material and choroid-plexus susceptibility, while brain organoid, animal, and microphysiological models support barrier injury, glial inflammatory responses, synaptic or myelin alterations, and mitochondrial/redox stress [20,22,38,64,66]. ATP, adenosine triphosphate; BBB, blood–brain barrier; BCSFB, blood–cerebrospinal fluid barrier; CNS, central nervous system; CSF, cerebrospinal fluid; ICAM-1, intercellular adhesion molecule 1; IL-1β, interleukin-1 beta; IL-6, interleukin 6; PASC, post-acute sequelae of SARS-CoV-2 infection; ROS, reactive oxygen species; SARS-CoV-2, severe acute respiratory syndrome coronavirus 2; TNF-α, tumor necrosis factor alpha; VCAM-1, vascular cell adhesion molecule 1; vWF, von Willebrand factor.

**Figure 4:**
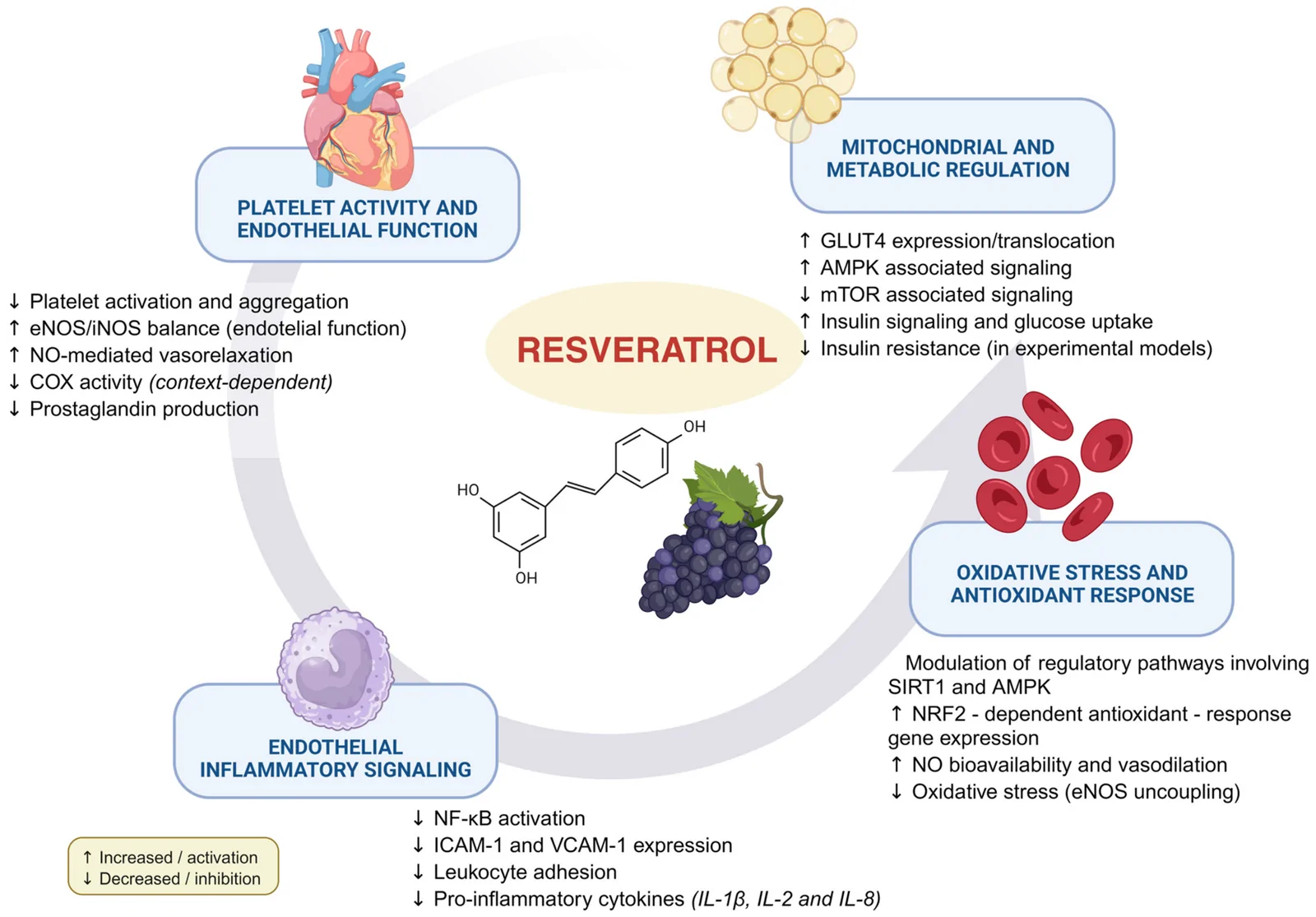
Experimentally reported resveratrol signaling domains relevant to neurovascular and inflammatory research. The figure summarizes molecular domains in which resveratrol has produced measurable effects in defined experimental systems. In primary endothelial studies, resveratrol increased Nrf2-responsive gene expression and reduced oxidant stress [77], increased Krüppel-like Factor 2 (KLF2) through SIRT1-associated signaling [78], and reduced TNF-α-induced inflammatory-factor expression through a cAMP–PKA–AMPK–SIRT1-associated autophagy pathway [4]. In oxidant-challenged endothelial cells, resveratrol reduced mitochondrial reactive oxygen species and apoptosis through AMPK–PGC-1α–ERRα–SIRT3 signaling [79]. ↑ Increased/activation; ↓ Decreased/inhibition. GLUT4, glucose transporter 4; ICAM-1, intercellular adhesion molecule 1; VCAM-1, vascular cell adhesion molecule 1; NO, nitric oxide; AMPK, AMP-activated protein kinase; mTOR, mechanistic target of rapamycin; COX, cyclooxygenase; SIRT1, sirtuin 1; Nrf2, nuclear factor erythroid 2-related factor 2; eNOS, endothelial nitric oxide synthase iNOS, inducible nitric oxide synthase; NFkB, nuclear factor kappa-light-chain-enhancer of activated B cells; IL-1β, interleukin-1 beta; IL-2, interleukin 2; IL-8, interleukin 8; PGC-1α, Peroxisome proliferator-activated receptor gamma coactivator 1-alpha; ERRα, estrogen-related receptor alpha. Nrf2 is a transcription factor, SIRT1 is an NAD^+^-dependent deacetylase, and AMPK is a protein kinase; these signaling regulators should not be classified as antioxidant enzymes.

**Figure 5:**
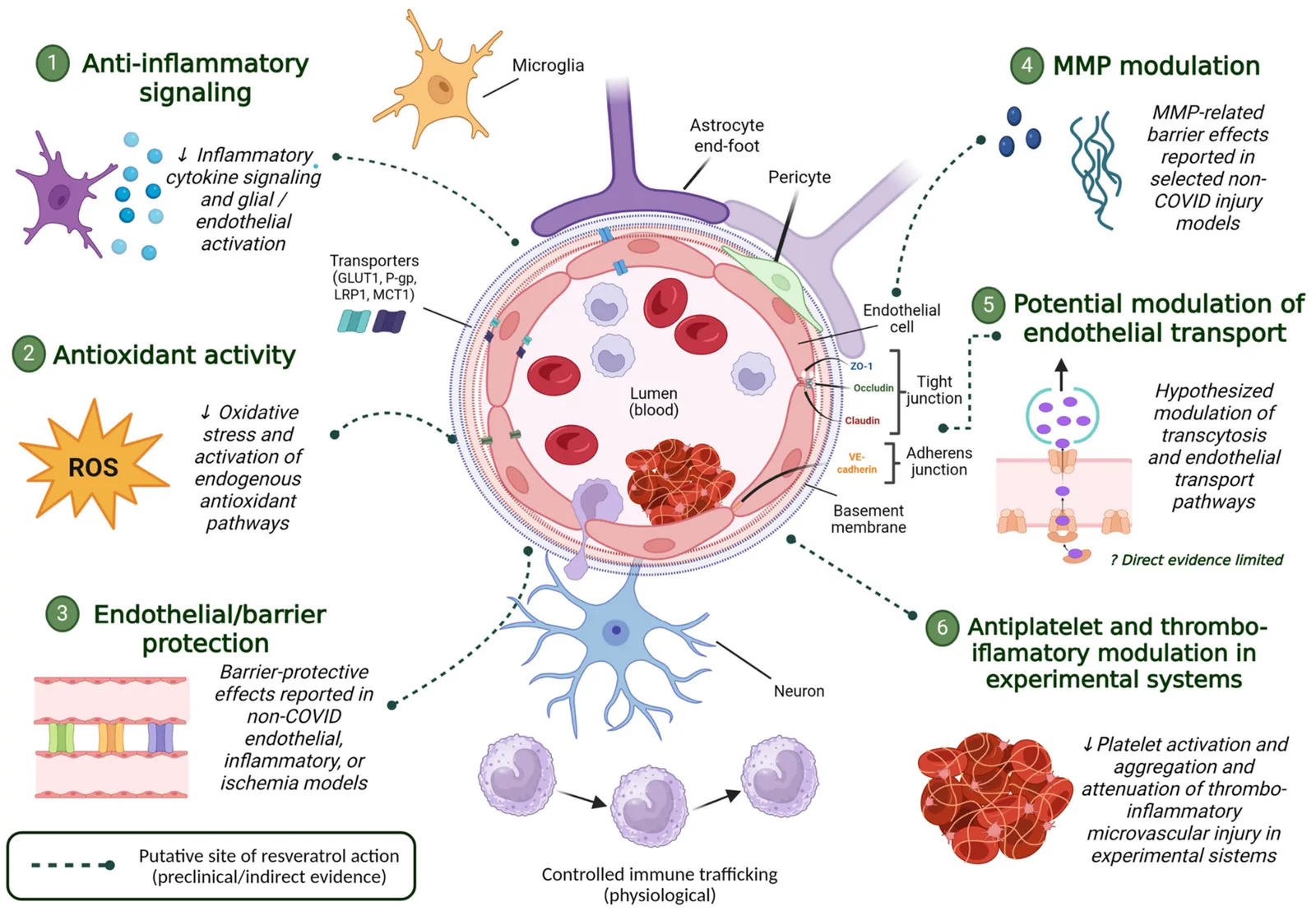
Potential sites of resveratrol action within the neurovascular unit. The schematic summarizes preclinical and indirect evidence that resveratrol can modify inflammatory signaling, oxidative stress, endothelial activation, barrier-associated readouts, matrix remodeling, and platelet activation. Primary studies have reported Nrf2-associated antioxidant effects in endothelial cells and mice, SIRT1/KLF2-associated endothelial signaling, and AMPK/SIRT1-associated reduction in TNF-α-induced endothelial inflammatory responses [4,77,78]. Barrier and matrix metalloproteinase-related effects derive primarily from non-COVID-19 injury models, whereas direct evidence for modulation of endothelial transport or transcytosis remains limited. Green dashed connectors indicate putative sites of resveratrol action supported predominantly by preclinical or indirect evidence derived from in vitro, ex vivo, and animal studies, frequently in models unrelated to SARS-CoV-2 infection. ↑ ↓ Decreased/inhibition. GLUT1, glucose transporter 1; LRP1, low-density lipoprotein receptor-related protein 1; MCT1 monocarboxylate transporter 1; MMP, matrix metalloproteinases; P-gp, P-glycoprotein; ROS, reactive oxygen species; SIRT1, Sirtuin 1; TNF-α, Tumor necrosis factor alpha; VE-cadherin, vascular endothelial cadherin.

**Figure 6:**
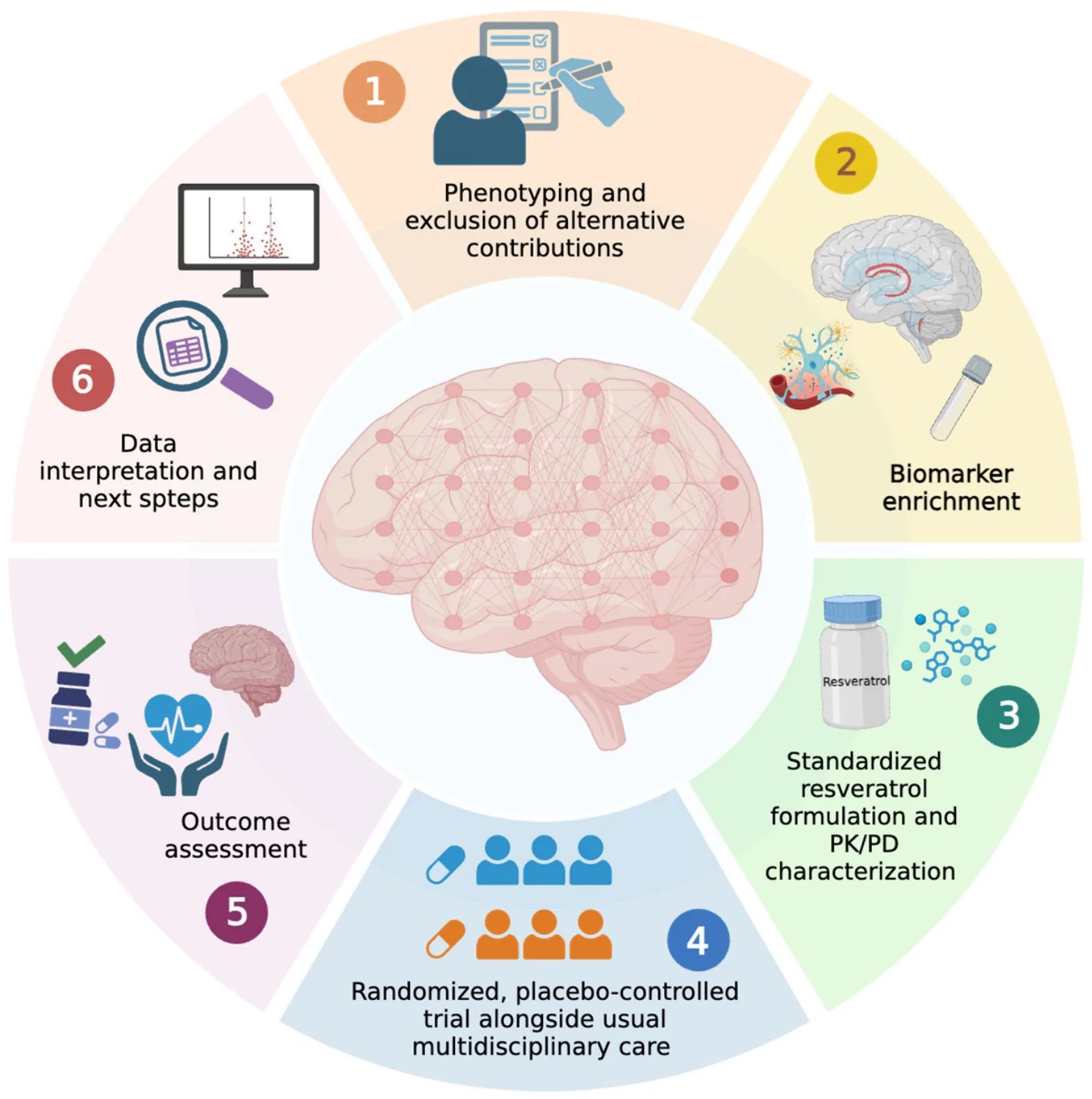
Proposed early-phase clinical-evaluation pathway for resveratrol as a research adjunct in post-COVID-19 cognitive dysfunction. The pathway comprises six sequential components. (1) Objective cognitive phenotyping, functional assessment, and evaluation of alternative or coexisting contributors guide participant selection [105]. (2) Neurovascular and inflammatory measures can serve as prospectively defined exploratory enrichment and target-engagement variables because blood–brain barrier and systemic inflammatory abnormalities have been identified in a subgroup with long-COVID-19 cognitive symptoms [37]. (3) Formulation-specific pharmacokinetic and pharmacodynamic assessment includes parent-resveratrol and metabolite measurements, since oral parent-compound exposure is strongly shaped by rapid metabolism [80]. (4) The randomized comparison evaluates resveratrol plus usual multidisciplinary care against matched placebo plus usual care. (5) Prespecified outcomes include objective cognition, everyday function, safety, exposure, and mechanism-matched target-engagement measures. (6) Integrated clinical, cognitive, functional, safety, pharmacokinetic, and biomarker results inform the decision to proceed to a larger confirmatory trial. PD, pharmacodynamics; PASC, post-acute sequelae of SARS-CoV-2 infection; PK, pharmacokinetics.

**Table 1:** Evidence hierarchy for interpreting mechanisms and interventions in post-COVID-19 cognitive dysfunction.

Evidence Category	Supported Inference
Direct human evidence	Association of a biomarker or intervention with cognitive outcomes in a defined post-COVID-19 cohort.
Indirect human evidence	Safety, pharmacokinetics, or biological plausibility from acute COVID-19 or other cognitive disorders.
Animal and organoid evidence	Cellular mechanisms, tissue vulnerability, and target selection.
In vitro/computational evidence	Target engagement hypotheses and concentration–response relationships.

**Table 2:** Effects of purified-resveratrol and grape-derived extracts in disease-model studies in non-COVID-19 neurological research.

Neurodegenerative Pathology/Model	Pathophysiology	Resveratrol Effects	Relevant Observations
Alzheimer [81,83,84,88]	Accumulation of β-amyloid (Aβ) plaques, tau protein hyperphosphorylation, OS, mitochondrial dysfunction, synaptic loss, and neuroinflammation mediated by microglia and astrocytes.	Wang et al. (2006) reported that Cabernet Sauvignon promoted non-amyloidogenic APP processing and reduced Aβ neuropathology in Tg2576 mice [83]. Porquet et al. (2013) found that dietary resveratrol reduced amyloid burden and tau hyperphosphorylation in SAMP8 mice [84]. In patients with AD, Turner et al. (2015) reported an attenuated decline in CSF Aβ40 concentrations [81]. In a retrospective analysis of a subset from the same trial, Moussa et al. (2017) found reduced CSF MMP-9 and increased CSF IL-4, macrophage-derived chemokine, and FGF-2 compared with placebo [88].	Wang et al. (2006) observed less spatial memory deterioration in Tg2576 mice receiving Cabernet Sauvignon; this effect cannot be attributed specifically to resveratrol [83]. Porquet et al. (2013) reported reduced cognitive impairment in SAMP8 mice receiving dietary resveratrol [84]. Turner et al. (2015) conducted a 52-week phase II RCT in 119 patients with mild-to-moderate AD, with doses escalating to 2 g/day; resveratrol and its major metabolites were detected in CSF [81]. Moussa et al. (2017) analyzed samples from 38 participants in that trial [88].
Parkinson [85]	Degeneration of dopaminergic neurons in the substantia nigra, mitochondrial dysfunction, α-synuclein aggregation, oxidative stress and neuroinflammation.	It suppressed rotenone-induced ROS production, inhibited COX and NADH oxidase, restored mitochondrial membrane potential, and activated the SIRT1 and AMPK signaling pathways.	It protected against neuronal apoptosis in human SH-SY5Y neuroblastoma cells exposed to rotenone toxicity. Authors did not establish efficacy in human patients.
Amyotrophic lateral sclerosis [86]	Progressive degeneration of upper and lower motor neurons associated with OS, mitochondrial dysfunction, glutamate excitotoxicity and neuroinflammation.	Delayed symptom onset preserved upper and lower motor neuron function and increased both overall survival and motor neuron survival. The effects were associated with SIRT1/AMPK signaling.	Study in mice; evaluated in SOD1 transgenic mice. Treatment initiated at 8 weeks extended overall survival and rotarod performance.
Huntington’s disease [89,90]	CAG repeat expansion in the HTT gene produces huntingtin with an expanded polyglutamine tract, associated with transcriptional dysregulation and mitochondrial dysfunction.	It supports the restoration of PGC-1α, TFAM, and mitochondrial DNA in lymphoblasts, as well as changes in mitochondrial gene expression in YAC128.	In YAC128 transgenic mice, 28 days of resveratrol treatment improved motor coordination and motor learning and increased the expression of mitochondrial-encoded electron transport chain genes.
Multiple sclerosis [91,92]	Autoimmune demyelinating disease characterized by chronic neuroinflammation, axonal damage and immune-mediated destruction of myelin.	Fonseca-Kelly et al. (2012) reported that resveratrol reduced neuronal loss in chronic EAE without significantly suppressing inflammation [91]. Shindler et al. (2007) found that SRT501, a resveratrol formulation, attenuated retinal ganglion cell loss in experimental optic neuritis; inhibition by sirtinol supported the involvement of SIRT1 [92].	Neuronal protection occurred despite persistent inflammation in the models examined. In Shindler et al. (2007), intravitreal SRT501 preserved axonal transport without reducing clinical EAE scores or the incidence of optic neuritis [92].
Vascular dementia [87,93]	Cerebral microvascular damage, chronic cerebral hypoperfusion, endothelial dysfunction, OS, neuroinflammation, and disruption of the blood–brain barrier leading to neuronal loss and cognitive decline.	Inhibits NF-κβ and microglial activation. Ameliorates cerebral hypoperfusion and neuroinflammation. Antioxidant and anti-inflammatory effects; improvement of endothelial function; reduction in OS; activation of SIRT1 and AMPK signaling; enhancement of cerebral blood flow and protection against neuronal apoptosis.	In rodent models of permanent bilateral common carotid artery occlusion, pure resveratrol reduced hippocampal CA1 neuronal loss and improved cognitive performance.

Aβ, amyloid beta peptide; AMPK, AMP-activated protein kinase; APP, amyloid precursor protein; CAG, cytosine–adenine–guanine trinucleotide repeat; EAE, experimental autoimmune encephalomyelitis; HD, Huntington’s disease; HTT, huntingtin gene; NF-κB, nuclear factor kappa B; OS, oxidative stress; PGC-1α, peroxisome proliferator-activated receptor gamma coactivator-1 alpha; ROS, reactive oxygen species; SIRT1, sirtuin 1.

**Table 3:** Translational crosswalk from neuro-COVID-19 mechanisms to resveratrol evidence.

Candidate Domain	Directness of PASC Evidence	Primary Resveratrol Evidence	Next Test
Neurovascular inflammation and endothelial activation	Greene et al. (2024) reported BBB leakage, coagulation-related blood signatures, PBMC adhesion to brain endothelial cells, and serum-induced endothelial inflammation in long-COVID-19 cognitive impairment [37].	Chen et al. (2013) found reduced TNF-α-induced inflammatory-factor expression in HUVECs through cAMP–PKA–AMPK–SIRT1-associated autophagy [4].	Enrich for an endothelial/neurovascular phenotype; measure exposure, endothelial markers, BBB imaging where feasible, and cognitive outcomes.
Mitochondrial oxidative stress and NAD^+^-related signaling	Izadpanah et al. (2023) found altered NAD-biosynthetic and NAD-consuming gene expression in acute pulmonary models and datasets; direct brain/PASC cognitive evidence is unavailable [42].	Zhou et al. (2014) showed reduced mitochondrial ROS and apoptosis in oxidant-challenged HUVECs through AMPK–PGC-1α–ERRα–SIRT3 signaling [79].	Measure parent compound/metabolites, redox and NAD^+^-related markers, and their prespecified relation to cognition.
Glial inflammatory and inflammasome signaling	Soung et al. (2022) examined hamsters and post-mortem human tissue and found hippocampal microglial activation and IL-1β/IL-6 induction in cases of acute COVID-19 [60].	Tufekci et al. (2021) reported inhibition of LPS/ATP-activated NLRP3, cytokine production, pyroptosis, NF-κB signaling, and miR-155 in N9 microglial cells [97].	Treat this as a preclinical hypothesis; use validated inflammatory or glial proximal exploratory markers rather than infer microglial target engagement.
Acute viral replication	Persistent neuronal replication is not established as the general cause of PASC cognitive symptoms [64].	Pasquereau et al. (2021) and Ter Ellen et al. (2021) reduced coronavirus/SARS-CoV-2 replication in cell systems, including primary bronchial epithelial cultures [11,98].	Do not use antiviral activity as a PASC cognitive efficacy rationale; reserve it for acute-infection hypotheses.
Objective cognitive performance	PASC cognitive symptoms are heterogeneous and require objective phenotyping and assessment of competing contributors.	Turner et al. (2015) detected resveratrol and metabolites in CSF in Alzheimer’s disease but did not establish clinical efficacy [81].	Use a validated cognitive composite as the primary endpoint and position target-engagement measures as secondary or exploratory.

AMPK, AMP-activated protein kinase; ATP, Adenosine triphosphate; BBB, blood–brain barrier; cAMP, cyclic adenosine monophosphate; CNS, central nervous system; CSF, cerebral spinal fluid; ERRα, estrogen-related receptor alpha; HUVECs, human umbilical vein endothelial cells; IL-1β, Interleu-kin-1 beta; IL-6, Interleukin-6; LPS, Lipopolysaccharide; NAD^+^, nicotinamide adenine dinucleotide; NF-κB, nuclear factor kappa B; NLRP3, NOD-like receptor family pyrin domain-containing 3; PASC, post-acute sequelae of SARS-CoV-2 infection; PBMC, peripheral blood mononuclear cells; PGC-1α, peroxisome proliferator-activated receptor gamma coactivator 1-alpha; PKA, protein kinase A; ROS, reactive oxygen species; SIRT1, sirtuin 1; SIRT3, Sirtuin 3; TNF-α, Tumor necrosis factor alpha.

**Table 4:** Human studies of resveratrol that inform clinical development for PASC cognitive impairment.

Study	Population and Design	Exposure or Main Result	Relevance to PASC Trial
McCreary et al. (2022) [99]	Acute outpatient COVID-19; randomized, double-blind, placebo-controlled phase 2 trial; 100 evaluable participants.	Hospitalization: 2% with resveratrol versus 6% with placebo; emergency visits: 8% versus 14%; pneumonia: 8% versus 16%. All confidence intervals were wide, and outcomes were statistically similar.	Provides short-term outpatient tolerability and feasibility data only; it did not assess PASC or cognition.
Bastin et al. (2025) [100]	Acute COVID-19; randomized, double-blind, placebo-controlled trial; 44 participants, 750 mg/day for 10 days.	Reported lower CRP, IL-1β, TNF-α, fasting glucose, neutrophils, and platelet count, with higher lymphocytes versus placebo.	Short-term peripheral biomarker evidence; no neurocognitive, pharmacokinetic, or post-acute outcome.
Turner et al. (2015) [81]	Mild-to-moderate Alzheimer’s disease; 52-week randomized, placebo-controlled phase 2 trial; 119 participants.	Parent resveratrol and major metabolites were measurable in plasma and CSF; nausea, diarrhea, and weight loss were common; MRI showed greater brain-volume loss with resveratrol.	Informs high-dose CNS exposure and safety monitoring, not efficacy in PASC cognition.
Witte et al. (2014) [101]	Healthy overweight adults aged 50–75 years; 46 participants; 26-week randomized, double-blind trial. Intervention: 200 mg/day resveratrol combined with 320 mg/day quercetin.	Improved 30 min word retention (*p* = 0.038), hippocampal functional connectivity, HbA1c, and body fat versus placebo.	Signals in healthy aging are indirect; moreover, the use of a combined formulation (resveratrol + quercetin) precludes attributing observed effects exclusively to resveratrol.
Evans et al. (2017) [103]	Postmenopausal women aged 45–85 years; 80 randomized participants; 14-week placebo-controlled trial.	Cerebrovascular responsiveness increased 17%; verbal memory and overall cognition improved, and cognitive change correlated with cerebrovascular responsiveness.	Supports measurement of cerebrovascular outcomes but is indirect for PASC mechanisms.
Thaung Zaw et al. (2021) [104]	Postmenopausal women; 125 participants; 24-month randomized, placebo-controlled crossover study of 75 mg twice daily.	Reported improvement in overall cognitive performance together with cerebral-blood-flow velocity and cerebrovascular responsiveness. The clinical meaning depends on the prespecified cognitive measure, crossover comparison, and uncertainty around the estimate.	Longer-term indirect evidence in a distinct population; it cannot establish efficacy in PASC cognitive impairment.
Köbe et al. (2017) [102]	Patients with MCI; 26-week trial, 42 randomized, 40 completed the intervention (18 resveratrol 200 mg/day vs. 22 olive oil placebo); n = 30 in fMRI subsample (14 intervention, 16 placebo). Intervention: 200 mg/day resveratrol combined with 350 mg/day quercetin.	Increased resting-state functional connectivity between left hippocampus and prefrontal/parietal regions and reduced HbA1c but no significant difference in primary declarative memory performance (AVLT) vs. placebo.	Highlights a disconnection between biological/fMRI modulation and clinical cognitive outcomes in MCI; additionally, the combined formulation (resveratrol + quercetin) prevents isolating the therapeutic effect of resveratrol alone.

CNS, central nervous system; CSF, cerebrospinal fluid; CRP, C-reactive protein; HbA1c, Glycated hemoglobin A1c; IL-1β, Interleukin-1 beta; MRI, Magnetic resonance imaging; PASC, post-acute sequelae of SARS-CoV-2 infection; TNF-α, tumor necrosis factor alpha; AVLT, Auditory verbal learning test; MCI, mild cognitive impairment.

**Table 5:** Current management options for post-COVID-19 cognitive sequelae and the proposed research role of resveratrol.

Management Domain	Current Evidence and Role	Relationship to Resveratrol
Diagnostic assessment	Essential: define cognitive phenotype and identify sleep, mood, autonomic, cardiopulmonary, medication, or metabolic contributors.	Required before considering any adjunct; identifies plausible enrichment variables.
Cognitive rehabilitation	Pilot randomized evidence suggests possible self-reported benefit; objective benefit remains uncertain.	Could serve as background care; resveratrol should be tested as an add-on, not a substitute.
Cardiopulmonary/physical rehabilitation	May improve stress, depression, conditioning, and function in selected patients; cognitive benefit not established.	No evidence that resveratrol enhances rehabilitation response.
Symptom-directed pharmacotherapy	Off-label/indication-specific use may be appropriate; no established disease-modifying drug for PASC cognition.	Potential interaction and safety assessment is necessary; no proven combination.
Resveratrol	Preclinical rationale and limited acute-COVID-19 human data; no direct PASC cognition trial.	Research adjunct only, with standardized formulation, PK, target engagement, and cognitive endpoints.

PASC, post-acute sequelae of SARS-CoV-2 infection; PK, pharmacokinetics.

## Data Availability

No new data were created or analyzed in this study. Data sharing is not applicable to this article.

## Abbreviations

The following abbreviations are used in this manuscript:

ACE2	Angiotensin-converting enzyme 2
AD	Alzheimer’s Disease
ALS	Amyotrophic lateral sclerosis
AMP	Adenosine monophosphate
AMPK	AMP-activated protein kinase
APP	Amyloid precursor protein
ATG5	Autophagy-related protein 5
ATP	Adenosine triphosphate
ATP6V0C	ATPase H+ transporting V0 subunit C
AVLT	Auditory Verbal Learning Test
Aβ1–42	Amyloid beta peptide 1–42
BBB	Blood–brain barrier
BCSFB	Blood–cerebrospinal fluid barrier
BDNF	Brain-derived neurotrophic factor
BECN1	Beclin 1
CA1	Cornu ammonis 1 hippocampal subfield
CAG	Cytosine–adenine–guanine trinucleotide repeat
cAMP	Cyclic adenosine monophosphate
CCL11	C-C motif chemokine ligand 11
CD38	Cluster of differentiation 38
CNS	Central nervous system
COX	Cyclooxygenase
COVID-19	Coronavirus disease 2019
CREB	cAMP response element-binding protein
CREB-1	cAMP response element-binding protein 1
CRP	C-reactive protein
CSF	Cerebrospinal-fluid
CXCL	C-X-C motif chemokine ligand
DNA	Deoxyribonucleic acid
E	Envelope protein of SARS-CoV-2
EAE	Experimental autoimmune encephalomyelitis
Enos	Endothelial nitric oxide synthase
ERRα	Estrogen-related receptor alpha
fMRI	Functional magnetic resonance imaging
GCLC	Glutamate-cysteine ligase catalytic subunit
GLUT1	Glucose transporter 1
GLUT4	Glucose transporter type 4
HbA1c	Glycated hemoglobin A1c
HCoV-229E	Human coronavirus 229E
HD	Huntington’s disease
HMOX1	Heme oxygenase 1
HTT	Huntingtin gene
HUVECs	Human umbilical vein endothelial cells
ICAM-1	Intercellular adhesion molecule 1
IDH2	Isocitrate dehydrogenase 2
Ido1	Indoleamine 2,3-dioxygenase 1
IFITM3	Interferon-induced transmembrane protein 3
IFN-γ	Interferon gamma
IL-1β	Interleukin-1 beta
IL-2	Interleukin-2
IL-6	Interleukin-6
IL-8	Interleukin-8
iNOS	Inducible nitric oxide synthase
K18-hACE2	Keratin 18 promoter-driven human angiotensin-converting enzyme 2 transgenic mouse model
KLF2	Krüppel-like factor 2
LPS	Lipopolysaccharide
LRP1	Low-density lipoprotein receptor-related protein 1
MCT1	Monocarboxylate transporter 1
ME/CFS	Myalgic encephalomyelitis/chronic fatigue syndrome
MEF2	Myocyte enhancer factor 2
MEK5	Mitogen-activated protein kinase kinase 5
miR-134	MicroARN 134
miR-134-5p	MicroRNA-134-5p
miR-155	MicroRNA-155
miR-369-3p	MicroRNA-369-3p
miR-448	MicroRNA-448
miR-450a-5p	MicroRNA-450a-5p
miR-483-3p	MicroRNA-483-3p
miRNAs	MicroRNAs
MMP	Matrix metalloproteinase
MMP-2/9	Matrix metalloproteinases 2 and 9
MoCA	Montreal Cognitive Assessment
MRI	Magnetic resonance imaging
mtDNA	Mitochondrial DNA
mTOR	Mechanistic target of rapamycin
N9	Murine microglial cell line
NAD^+^	Nicotinamide adenine dinucleotide
NADH	Nicotinamide adenine dinucleotide, reduced form
NADPH	Nicotinamide adenine dinucleotide phosphate
NAMPT	Nicotinamide phosphoribosyl transferase
Naprt1	Nicotinate phosphoribosyl transferase 1
NF-κB	Nuclear factor kappa
NLRP3	NOD-like receptor family pyrin domain-containing 3
Nmnat1	Nicotinamide nucleotide adenylyl transferase 1
NO	Nitric oxide
NQO1	NAD(P)H quinone dehydrogenase 1
Nrf2	Nuclear factor erythroid 2-related factor 2
Nrk	Nicotinamide riboside kinase
NSP14	Nonstructural protein 14
NSP4	Nonstructural protein 4
NVU	Neurovascular unit
ORF 3a	Open reading frame 3a protein of SARS-CoV-2
ORF 9b	Open reading frame 9b protein of SARS-CoV-2
OS	Oxidative stress
P-gp	P-glycoprotein
PARP	Poly(ADP-ribose) polymerases
PARP10	Poly(ADP-ribose) polymerase family member 10
PARP14	Poly(ADP-ribose) polymerase family member 14
PARP9	Poly(ADP-ribose) polymerase family member 9
PARPs	Poly(ADP-ribose) polymerase
PASC	Post-acute sequelae of SARS-CoV-2 infection
PBMC	Peripheral blood mononuclear cells
PD	Pharmacodynamics
PGC-1α	Peroxisome proliferator-activated receptor gamma coactivator 1-alpha
PK	Pharmacokinetics
PKA	Protein kinase A
Q10	Coenzyme Q10
RCT	Randomized controlled trial
RNA	Ribonucleic acid
RNS	Reactive nitrogen species
ROS	Reactive oxygen species
S1	Spike protein subunit 1
SANRA	Scale for the Assessment of Narrative Review Articles
SARS-CoV-2	Severe acute respiratory syndrome coronavirus 2
SH-SY5Y	Human neuroblastoma cell line
siRNA	Small interfering RNA
SIRT1	Sirtuin 1
SIRT3	Sirtuin 3
SOD1	Superoxide dismutase 1
SOD2	Superoxide dismutase 2
Tg2576	Transgenic mouse model of Alzheimer’s disease
TGF-β	Transforming growth factor beta
TLR9	Toll-like receptor 9
TNF	Tumor necrosis factor
TNF-α	Tumor necrosis factor alpha
VCAM-1	Vascular cell adhesion molecule 1
VE-cadherin	Vascular endothelial cadherin
Vero	African green monkey kidney epithelial cell line
vWF	Von Willebrand factor
YY1	Yin Yang 1 transcription factor
ZO-1	Zonula occludens 1
